# Evidence gaps and diversity among potential win–win solutions for conservation and human infectious disease control

**DOI:** 10.1016/S2542-5196(22)00148-6

**Published:** 2022-08-03

**Authors:** Skylar R Hopkins, Kevin D Lafferty, Chelsea L Wood, Sarah H Olson, Julia C Buck, Giulio A De Leo, Kathryn J Fiorella, Johanna L Fornberg, Andres Garchitorena, Isabel J Jones, Armand M Kuris, Laura H Kwong, Christopher LeBoa, Ariel E Leon, Andrea J Lund, Andrew J MacDonald, Daniel C G Metz, Nicole Nova, Alison J Peel, Justin V Remais, Tara E Stewart Merrill, Maya Wilson, Matthew H Bonds, Andrew P Dobson, David Lopez Carr, Meghan E Howard, Lisa Mandle, Susanne H Sokolow

**Affiliations:** a Department of Applied Ecology, North Carolina State University, Raleigh, NC, USA; b National Center for Ecological Analysis and Synthesis, Santa Barbara, CA, USA; c Western Ecological Research Center, US Geological Survey at Marine Science Institute, University of California, Santa Barbara, CA, USA; d Department of Ecology, Evolution, and Marine Biology, University of California, Santa Barbara, CA, USA; e Bren School of Environmental Science and Management, University of California, Santa Barbara, CA, USA; f Department of Geography, University of California, Santa Barbara, CA, USA; g Division of Environmental Health Sciences, University of California, Berkeley, CA, USA; h Scripps Institution of Oceanography, University of California, San Diego, CA, USA; i School of Aquatic and Fishery Sciences, University of Washington, Seattle, WA, USA; j Wildlife Conservation Society, Health Program, Bronx, NY, USA; k Department of Biology and Marine Biology, University of North Carolina Wilmington, Wilmington, NC, USA; l Hopkins Marine Station, Stanford University, Pacific Grove, CA, USA; m Woods Institute for the Environment, Stanford University, Stanford, CA, USA; n Department of Epidemiology, Stanford University, Stanford, CA, USA; o Department of Biology, Stanford University, Stanford, CA, USA; p Department of Population Medicine and Diagnostic Sciences and Master of Public Health Program, Cornell University, Ithaca, NY, USA; q MIVEGEC, Université Montpellier, Centre National de la Recherche Scientifique, Institut de Recherche pour le Développement, Montpellier, France; r NGO PIVOT, Ranomafana, Madagascar; s Department of Biological Sciences, Virginia Tech, Blacksburg, VA, USA; t US Geological Survey, National Wildlife Health Center, Madison, WI, USA; u Department of Environmental and Occupational Health, University of Colorado School of Public Health, Aurora, CO, USA; v Centre for Planetary Health and Food Security, Griffith University, Nathan, QLD, Australia; w Coastal and Marine Laboratory, Florida State University, St Teresa, FL, USA; x Department of Global Health and Social Medicine, Harvard Medical School, Boston, MA, USA; y Ecology and Evolutionary Biology, Princeton University, Princeton, NJ, USA

## Abstract

As sustainable development practitioners have worked to “ensure healthy lives and promote well-being for all” and “conserve life on land and below water”, what progress has been made with win–win interventions that reduce human infectious disease burdens while advancing conservation goals? Using a systematic literature review, we identified 46 proposed solutions, which we then investigated individually using targeted literature reviews. The proposed solutions addressed diverse conservation threats and human infectious diseases, and thus, the proposed interventions varied in scale, costs, and impacts. Some potential solutions had medium-quality to high-quality evidence for previous success in achieving proposed impacts in one or both sectors. However, there were notable evidence gaps within and among solutions, highlighting opportunities for further research and adaptive implementation. Stakeholders seeking win–win interventions can explore this Review and an online database to find and tailor a relevant solution or brainstorm new solutions.

## Introduction

Ecosystem degradation can exacerbate infectious diseases that have long plagued humankind or cause novel pathogens to spillover from animals to humans.^1^, ^2^, ^3^, ^4^, ^5^, ^6^ By targeting connections between human infectious disease and the natural world, interventions might “ensure healthy lives and promote well-being for all” and “conserve life on land and below water”—two Sustainable Development Goals (SDGs).^7^, ^8^, ^9^, ^10^, ^11^, ^12^, ^13^, ^14^, ^15^, ^16^, ^17^ For example, putting tick collars on free-ranging dogs might reduce transmission of ticks and tick-borne disease from dogs to people and wildlife.^18^ Indeed, sustainable development practitioners worldwide are urgently seeking safe and effective cross-sector interventions that might prevent the next pandemic.^19^

Of course, no single win–win intervention will work in all contexts or solve all problems within complex socioecological systems.^20^ Interventions that improve some outcomes for human health and ecosystems might even cause collateral impacts in other sectors, creating complex trade-offs among SDGs.^16^, ^21^, ^22^ Tasked with choosing an optimal intervention for any given problem and socioecological context, practitioners need to know about the available intervention options and how they can be compared. In the event that no existing intervention is suitable, practitioners will need to know how to identify and evaluate new intervention options.

Unfortunately, the information needed to identify, implement, and evaluate win–win interventions that prevent or control human infectious diseases tends to be scarce, inconsistent, and unconsolidated.^23^ For instance, among conservation intervention studies that reported human wellbeing benefits, fewer than 2% considered health-specific outcomes and only a subset of those considered emerging or endemic infectious diseases.^24^, ^25^ Furthermore, existing studies are scattered across siloed disciplines that use different research methodologies, measure different outcomes, and publish in different journals. Navigating this dispersed evidence landscape would be prohibitively time consuming for practitioners interested in implementing win–win interventions.

**Figure 1:**
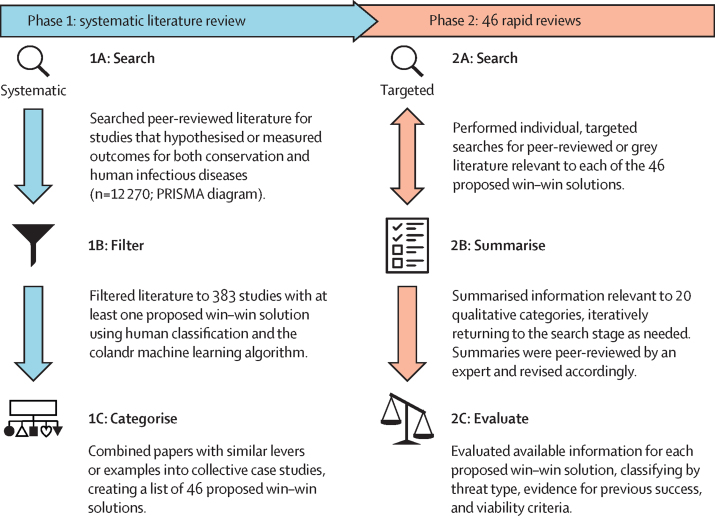
Subject-wide evidence synthesis The PRISMA diagram is shown in the appendix (p 14).

**Figure 2:**
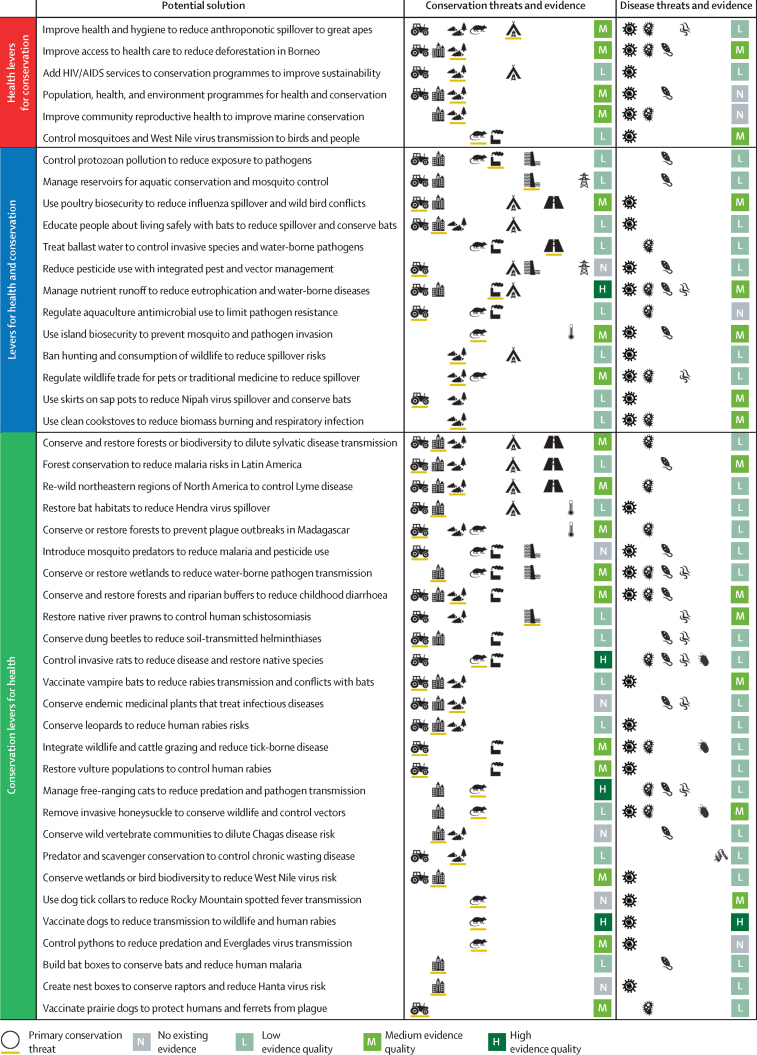
A menu of 46 potential solutions for advancing conservation goals and controlling human infectious diseases N denotes none (defined as hypotheses and anecdotes), L denotes low (defined as some supporting studies with moderate to major gaps, inconsistency, or low applicability), M denotes medium (defined as several lines of evidence that are mostly consistent and applicable), and H denotes high (defined as diverse, consistent, and highly applicable evidence that leaves little to no uncertainty regarding the outcome).

To facilitate timely, evidence-based decision making, we review existing evidence regarding win–win solutions that aim to simultaneously reduce human infectious disease burden and advance conservation goals. We use the terms intervention and solution interchangeably because there are growing policy initiatives for enacting nature-based solutions.^8^ To find, evaluate, and synthesise evidence from win–win solutions, we used a subject-wide evidence synthesis (figure 1). This two-phase method uses a systematic literature review to identify a landscape of possible interventions and then each intervention is explored using an individual, targeted rapid review.^26^, ^27^, ^28^, ^29^ This process allowed us to create a menu of 46 proposed solutions ranging from local to international scales, including individual information summaries (figure 2). Stakeholders can explore these examples to find and tailor potential solutions to meet their needs or brainstorm new solutions. Potentially viable solutions that achieve specific goals within resource constraints can be identified and evaluated using general criteria that we derived from synthesising information across the 46 solutions. Finally, we highlight evidence gaps within and among solutions that could be important targets for future research and implementation.

## Widespread and diverse potential solutions

**Figure 3:**
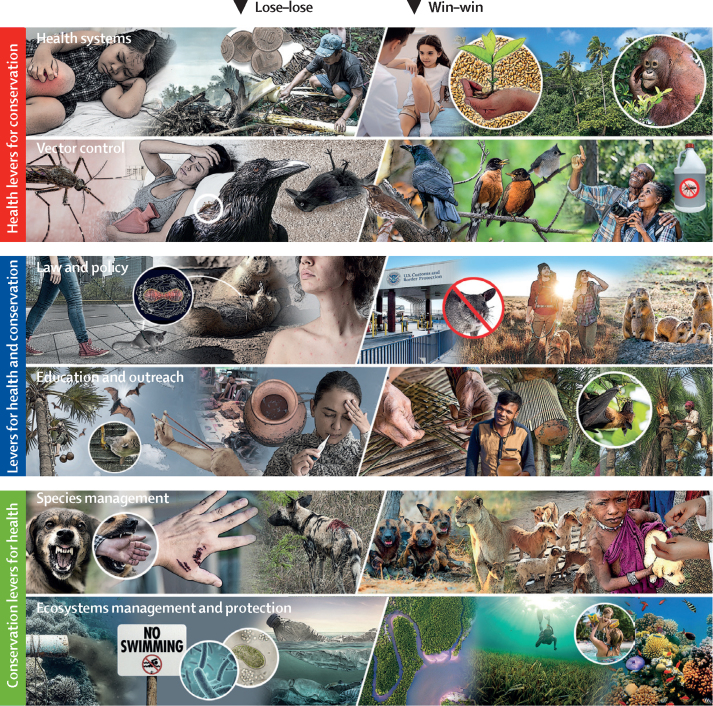
Six lose–lose scenarios that could be improved with win–win solutions This figure was commissioned from artist Hiram Henriquez, and all photographs were used under creative commons licences or purchased with commercial licences (ie, iStockphoto). Photographs of palms and sap collection pots were used with permission from Fernando Garcia and Nazmun Nahar.

**Figure 4:**
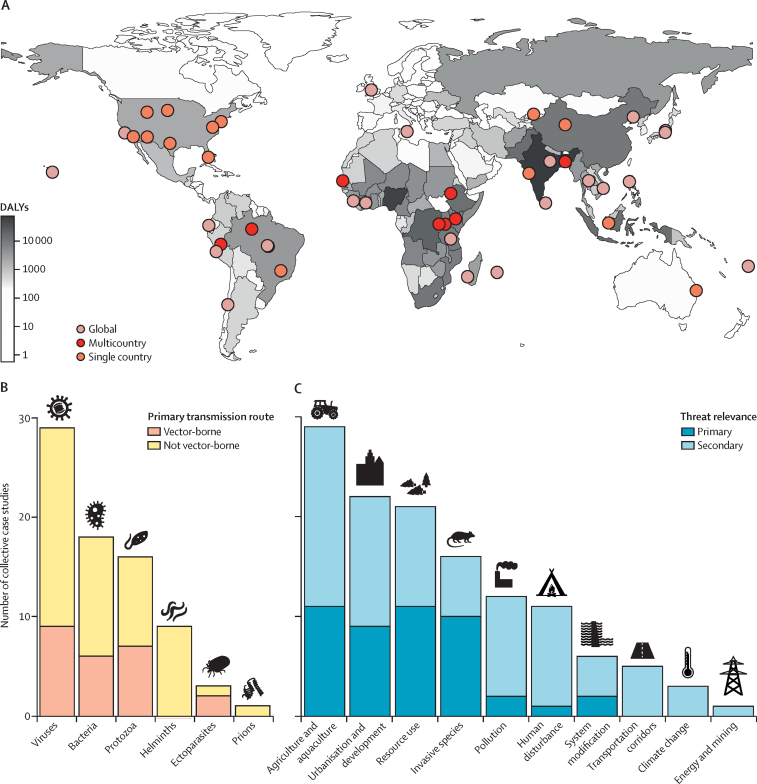
Solutions are widespread and diverse (A) The solutions covered all continents (except Antarctica), including countries with high and low burdens of infectious diseases, as measured by total DALYs and reported by WHO in 2018.^30^ (B) The solutions covered most pathogen taxa (except fungi) and transmission modes. (C) Seven International Union for Conservation of Nature threat classes were considered the primary conservation threat addressed by at least one solution, whereas transportation, climate change, and energy and mining were only secondary conservation threats addressed by any solution. DALYs=disability-adjusted life-years.

The 46 potential solutions addressed diverse threats, collectively covering all continents (except Antarctica), most major pathogen groups (except fungi), and most conservation threat classes defined by the International Union for Conservation of Nature (IUCN; except geological events and other; figures 3, 4).^31^ Most solutions addressed multiple threats for health and conservation (eg, multiple pathogen species and multiple IUCN threat classes). The 46 potential solutions also covered numerous intervention types and targets, ranging from vaccinating vampire bats against rabies in Peru^32^ to establishing sustainable harvesting programmes for medicinal plant species in Tanzania.^33^ Potential solutions were diverse because the problems that they addressed were diverse.

Most solutions addressed pathogens with environmentally mediated transmission, such as vector-borne diseases and zoonotic diseases transmitted from animals to people (figure 4), mirroring the strong focus on these diseases in the One Health, Planetary Health, and EcoHealth fields.^9^, ^11^, ^34^ For example, WHO and other international organisations support the expansion of training for integrated pest and vector management globally, because these management techniques might reduce total pesticide use and control crop pests and disease vectors, such as mosquitoes. Environmentally mediated diseases such as these have probably been the easiest entry points for cross-sector solutions due to obvious underlying links between human health and ecosystems.

Only a few potential solutions addressed diseases without environmentally mediated transmission, such as HIV/AIDS and pneumonia. For example, people with poor health due to HIV/AIDS or other diseases are more likely to use easier and more destructive fishing practices in communities near Lake Victoria in Africa, so treatment and prevention programmes might support both human health and aquatic conservation.^35^ Focusing on these understudied links between ecosystems and directly transmitted and chronic human diseases might yield additional solutions for advancing conservation and health.

In addition to addressing diverse health threats, the potential solutions also addressed all IUCN conservation threats related to anthropogenic activities (figure 4C). Solutions related to land use change—agriculture and aquaculture (n=29 collective case studies) and urbanisation and development (n=22)—were most common, probably because land use change is a leading driver of biodiversity declines^36^ and disease spillover.^6^, ^11^, ^37^, ^38^ Only a few solutions addressed transportation infrastructure, energy and mining, and climate change, and these were never addressed as the primary threat.

We did not include a collective case study in which climate change was the primary conservation threat and global emission reduction was the solution (appendix pp 3–4) because the numerous health and conservation outcomes that could be achieved by global emission reduction^9^, ^10^ have yet to be measured. Climate change is expected to become a more urgent threat over time,^7^ so there is a clear need for actionable targeted solutions related to climate change.

The conservation and health threats targeted by potential solutions spanned from subnational extents within single countries to multiple countries within a continent to global applicability (figure 4A). For example, vaccinating prairie dogs to reduce plague risk for endangered black-footed ferrets and humans applies subnationally in Wyoming, South Dakota, Montana, and Arizona (USA);^39^ whereas forest conservation to reduce human malaria might be relevant to multiple countries in Latin America, Africa, and Asia.^40^, ^41^ The potential solutions included targets in low-income and middle-income countries with high burden of environmentally mediated human infectious diseases and high-income countries with lower infectious disease burden and higher research efforts. Ultimately, one or more potential solutions probably exist for all countries, but the set of relevant solutions that apply to any country could be expanded by future efforts to scale or translate existing solutions to new locations.

There were 27 potential solutions that involved implementing classic conservation interventions that have health benefits (ie, conservation levers for health; figure 2), which are sometimes called nature-based solutions or ecological levers for health.^8^, ^42^, ^43^ One common conservation intervention type was species management, including controlling or eradicating invasive honeysuckle to reduce negative impacts on native vertebrates and reduce vector populations^44^ and reintroducing native prawns extirpated by dams to help control snails that transmit human schistosomiasis.^45^ Another common conservation intervention type was land and water management or protection, such as conserving or restoring wetlands to increase biodiversity and reduce water-borne diarrhoeal diseases.^46^ Together, these 27 potential solutions are the most comprehensive list to date of conservation solutions that might reduce human infectious diseases, an important subsector within the global focus on conservation solutions that improve general human wellbeing.^8^, ^9^, ^11^, ^42^

Six potential solutions involved public health interventions that have conservation benefits (ie, health levers for conservation). For example, health system strengthening or family planning and reproductive health programmes—including population, health, and environment programmes in many countries^47^—have reduced illegal logging and deforestation;^48^, ^49^ improved coral and mangrove conditions in marine environments;^50^ and improved community participation in, or approval of, conservation initiatives.^51^ There were also several solutions that used insect vector control to reduce vector-borne disease risk for people and wildlife.^52^, ^53^ These health interventions were often supported by scarce evidence, either because there were not enough resources dedicated to monitoring and evaluation or interventions implemented by the health sector did not quantify ecosystem or conservation outcomes. Therefore, we expect that more health interventions that advance conservation goals could be developed and evaluated through increased collaboration between conservation and health organisations.

Finally, 13 potential solutions acted through interventions that were not specific to public health or conservation, but affected both sectors (ie, levers for health and conservation). Many of these were policies regarding the food–energy–water nexus,^54^ such as regulating protozoan pollution,^55^ reducing antibiotic use in aquaculture,^56^ reducing nutrient pollution and eutrophication associated with agriculture,^57^ and implementing ballast water treatment protocols to prevent invasive pathogens and wildlife from moving among ports.^58^ There were also outreach, education, or livelihood interventions such as teaching people how to live safely with bats that might be virus reservoirs,^59^ protecting tree sap collection pots from bat contamination using bamboo skirts,^60^, ^61^ and replacing wood-burning stoves with cleaner cookstoves to reduce deforestation and smoke-related pneumonia.^62^ However, livelihood-focused interventions were rare, so future efforts might discover more interventions that primarily target poverty and inequalities (SDGs 1 and 10) and that have downstream benefits for health and conservation (SDGs 1, 14, and 15).

Examples of each lever type are illustrated in figure 3. The figure illustrates (1) health systems that provide affordable health care in the Indonesian portion of the island of Borneo reduce human disease burden and illegal logging done to pay for health care (health lever); (2) vector control is a public health intervention that might also benefit biodiversity, as in the case of North American birds susceptible to the West Nile virus (health lever); (3) law and policy interventions that ban importation of non-native wildlife reservoirs (eg, pouch rats) prevent spillover to humans and native wildlife (lever for health and conservation); (4) education and outreach empower people to live safely with bats, reducing zoonotic spillover risk (eg, Nipah virus; lever for health and conservation); (5) species management, such as vaccinating or sterilising free-ranging domestic dogs, reduces rabies transmission to humans and African carnivores (conservation lever); and (6) ecosystem management interventions, such as restoring wetland vegetation, reduce the survival of human and wildlife pathogens in the environment while restoring wildlife habitat (conservation lever).

**Figure 5:**
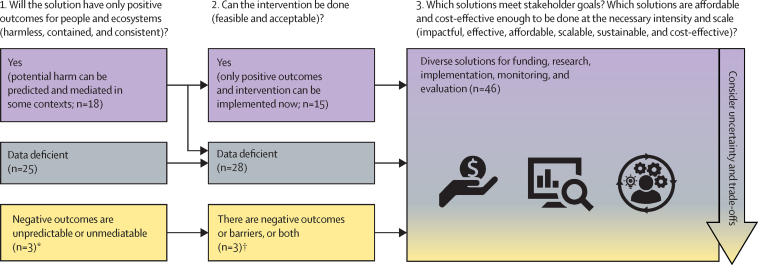
Viable solutions can be identified and evaluated by 11 criteria We evaluated five criteria to determine whether solutions were harmless, contained, consistent, feasible, and acceptable in predictable contexts given the currently available evidence or whether these evidence were data deficient for the specified criteria. Stakeholders can evaluate six other criteria on the basis of priorities and resource constraints (impactful, effective, affordable, scalable, sustainable, and cost-effective). *Three potential solutions had evidence for trade-offs that were unmitigable or unpredictable and were categorised as not harmless, not consistent, or both. All solutions were categorised as contained or data deficient for the contained criterion. †None of the potential solutions had evidence for unmediatable barriers to implementation (not feasible or not acceptable).

The diversity among potential solutions is promising because “there is no one-size-fits-all approach for One Health implementation”.^11^ The 46 examples we described here cover many context-specific health and conservation threats; therefore, stakeholders might be able to adapt one of these solutions to meet their needs. In cases where none of the potential solutions are relevant, stakeholders could design new solutions to meet specific goals within their resource constraints. To determine whether any given solution will be viable for a given context, stakeholders can evaluate each solution using the 11 viability criteria described below (harmless, contained, consistent, feasible, acceptable, impactful, effective, affordable, scalable, sustainable, and cost-effective; figure 5; appendix pp 7–8).

## Identifying and minimising trade-offs

To evaluate potential trade-offs caused by a given intervention, we suggest considering three viability criteria (figure 5): (1) harmless solutions are not expected to harm non-target aspects of human wellbeing for some people while attempting to help others; (2) contained solutions are not expected to have negative, collateral effects on non-human targets, or else potential collateral effects could be avoided or fully mitigated; and (3) consistent solutions are expected to have only positive outcomes for their intended conservation and human infectious disease control targets in predictable contexts (ie, no known negative outcomes). Two investigators used these definitions and the available evidence to determine whether each potential solution met, did not meet, or was data deficient for the three criteria (appendix pp 6–7). Data limitations often made it difficult to decide whether a solution involved substantial trade-offs, but existing evidence showed that 18 solutions could be harmless, contained, and consistent under some contexts (figure 5).

Context dependency was common among the 46 potential solutions. For example, introducing invasive predators that consume larval mosquitoes to control malaria has often negatively impacted ecosystems (eg, violating the contained criterion),^63^ but at least in some contexts (eg, native predators), mosquito predators are expected to only have positive outcomes for ecosystems (ie, meeting the contained criterion). However, for other solutions, we could not identify a mediating context. For example, correlational studies suggest that forest cover and host biodiversity impact competent host abundance in ways that can reduce human Lyme disease risk in North America (also known as the dilution effect).^64^ Yet in this complex system, forest cover or host biodiversity have also been associated with unexpected amplification of Lyme disease risk;^65^ thus, how forest conservation or restoration interventions would impact human health remains unclear (ie, this solution violates the consistent criterion). Future research or innovation might identify specific scales and circumstances in which this solution does no harm, but at present, it risks causing unpredictable harm to some people in certain contexts.

Similarly to most solutions, those that address pathogen spillover from wildlife to humans had data gaps regarding trade-offs. For example, wildlife trade is a conservation threat and pathway for spillover from wildlife.^66^, ^67^, ^68^, ^69^ However, in several African countries, past bans on all wildlife hunting and consumption sometimes created food insecurity, illegal markets, and distrust in health authorities.^21^, ^70^, ^71^, ^72^ Bans developed in response to the COVID-19 pandemic might be similarly problematic.^73^, ^74^ This evidence shows that wildlife trade bans can cause harm when they affect subsistence hunting and consumption, but might be safe and feasible in other specific contexts. For example, many existing national and international wildlife pet trade restrictions aim to conserve wildlife (eg, the Convention on International Trade in Endangered Species), and there is increasing, but not universal, public support for bans or restrictions on luxury commercial wildlife trade in Asia due to the 21st century coronavirus outbreaks.^75^ If successful, restrictions and bans that target the multibillion-dollar commercial wildlife trade could prevent multitrillion-dollar pandemics.^16^, ^76^ However, whether and when these policies will be successful is still unclear, including whether they will favour illegal markets or erode support for conservation. Efforts are urgently needed to identify contexts in which negative impacts are mediated, conservation is advanced, and spillover risks are reduced.

## Achieving socially acceptable and feasible solutions

Two criteria can be used to determine whether solutions are immediately achievable (ie, the feasible and acceptable criteria; figure 5). Feasible solutions could be successfully implemented given existing technology and sufficient resources, and socially acceptable solutions are supported by stakeholders who are affected by the intervention or could be made acceptable to stakeholders. For example, the livestock medication diclofenac caused substantial vulture population declines in India, which could have contributed to the increase in free-ranging dogs and human rabies risk.^77^ Therefore, diclofenac was banned in India and the surrounding nations, an intervention that was achievable because there were acceptable alternative veterinary drugs that could replace diclofenac and these were not toxic to vultures.^77^ As this example illustrates, some of the 46 potential solutions have already been successfully implemented on national or multinational scales.

However, most potential solutions were data deficient for feasibility or social acceptability. For example, two potential solutions involve the effort to broadly re-wild North America with top carnivores to control infectious diseases in wild herbivores and possibly humans.^78^ These potential solutions face opposition from some stakeholders (eg, ranchers and hunters), and whether these solutions would lead to net reductions in disease risk and whether they can be made socially acceptable in places in which the intervention would impact disease transmission the most remains unclear. Indeed, solutions that involved changing peoples’ lifestyles and cultures were often data deficient for acceptability, which highlights a clear need for social science and implementation research to evaluate cross-sector solution viability, as outlined by the Organisation for Economic Co-operation and Development.^79^

## Impactful and effective solutions achieve stakeholders’ goals

**Figure 6:**
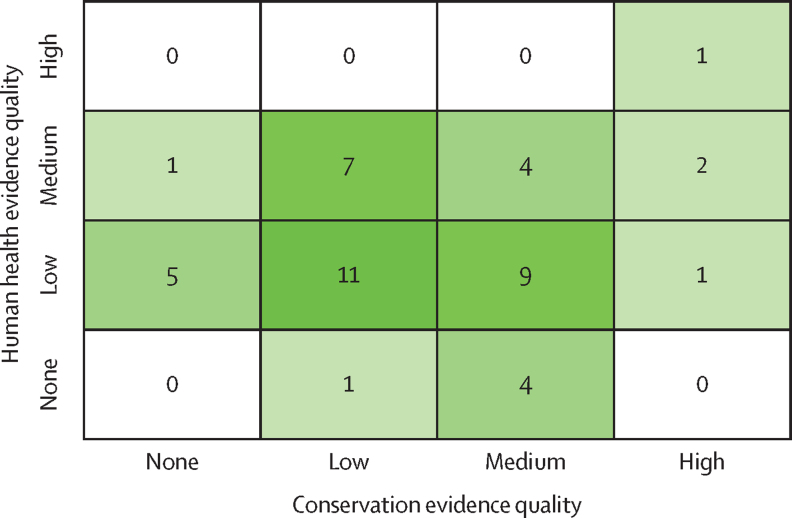
Most solutions had evidence gaps Each cell contains the number of solutions that had a given composite evidence quality score on the basis of evidence diversity, consistency, and applicability (none is defined as hypotheses and anecdotes; low quality evidence is defined as some supporting studies with moderate to major gaps, inconsistency, or low applicability; medium is defined as several lines of evidence that are mostly consistent and applicable; and high is defined as diverse, consistent, and highly applicable evidence that leaves little to no uncertainty regarding the outcome). The green colour is used to emphasise that most solutions were supported by low or medium evidence for conservation or health outcomes.

Whether a given solution meets stakeholders’ goals can be evaluated using two criteria (ie, the impactful and effective criteria). Impactful solutions have the potential to meet stakeholders’ quantitative goals (ie, effect magnitude and clinical relevance) and effective solutions can successfully achieve the desired outcomes. For example, building nest boxes to increase local predatory bird populations is proposed to control the rodent species that are reservoirs for hantaviruses.^80^ This solution is a proposed ecological lever for health.^42^ However, there is no evidence that nesting sites are scarce so the intervention might only redistribute non-threatened wildlife populations in ways that benefit humans, creating little value for stakeholders with strong conservation priorities (ie, not impactful for conservation). There is also scarce evidence that this solution can successfully reduce human disease burdens (ie, might not be effective for human health). We did not quantify how impactful each potential solution was because there was no common system available to rank impacts given the diverse methods and metrics used across relevant literature. However, we did qualitatively assess how effective each solution was, as evidenced by previous success in achieving proposed goals (figure 6; appendix pp 5–7).

We categorised existing evidence quality for each potential solution using a modified Bridge Collaborative rubric^81^ with three categories (evidence types and diversity, evidence consistency, and evidence applicability; appendix pp 5–7, 15). We then combined these three categories into one composite score for overall evidence quality for health outcomes and one score for conservation outcomes (Figure 2, Figure 6). No evidence indicated that cases were supported only by hypotheses and anecdotes; low quality evidence indicated that there were few supporting studies with moderate to major evidence gaps, unexplained inconsistency, or low applicability; medium quality indicated that there were several lines of evidence that were mostly consistent and applicable, and inconsistency could be explained; and high quality indicated diverse evidence types, usually including an intervention study, that yielded consistent and applicable results and left little to no uncertainty regarding the outcome. The resulting composite evidence quality scores highlight which solutions have had demonstrable success for health and conservation outcomes and which still have evidence gaps.

There were seven solutions that already had medium to high evidence quality for conservation and human health success (figure 6). For example, vaccinating dogs and wild carnivores to reduce rabies transmission among dogs, wildlife, and people was supported by high evidence quality for both outcomes (including successful intervention programmes).^82^, ^83^ Most solutions had higher evidence quality regarding effectiveness for conservation than for health. For example, controlling invasive rats, brushtail possums, and cats can conserve endemic species,^84^, ^85^, ^86^ especially on islands. There are also studies linking invasive species control and human infectious disease burden,^87^, ^88^ but the evidence types and diversity are low. Similarly, although forest restoration and conservation have well established benefits to ecosystem structure and function^89^, ^90^ and several correlational studies link upstream forest cover to reduced childhood diarrhoea risk downstream,^91^ none of these are intervention studies. Although evidence for these solutions could still be improved, these examples show that effective cross-sector solutions exist.

There were 17 potential solutions that had low overall evidence quality due to low evidence diversity, applicability, or consistency that was difficult to explain for one or both outcomes. For example, two observational studies^92^, ^93^ (scarce evidence diversity) quantified high leopard predation rates on free-ranging domestic dogs, an important disease reservoir for rabies near Mumbai (India).^92^ Leopard conservation might reduce dog rabies, and thus human rabies risk, but predation or culling of dogs could also counterintuitively increase rabies in dog reservoir populations (ie, potentially not consistent).^94^ In another example, regulating drawdown rates for water reservoirs created by dams might restore aquatic communities and reduce larval mosquito survival, thus reducing human malaria risk near dams.^95^ However, the existing evidence did not come from countries with high malaria burden (ie, low applicability). Additionally, integrating wild grazing animals on land parcels used for grazing cattle (frequently treated for ticks) might increase forage availability for wildlife and reduce tick abundance on land parcels,^96^ which in turn might affect human disease risk, but tick-borne disease incidence in humans has never been measured for this solution (ie, low applicability). As these examples illustrate, many promising solutions had some supporting evidence, but most solutions had notable data gaps (figure 5).

## Optimal solutions achieve goals within resource constraints

In addition to goals (ie, impactful and effective criteria), stakeholders also have resource constraints. Resources determine which solutions can be implemented at the necessary scales and intensities to achieve the desired outcomes (eg, the affordable, scalable, and sustainable criteria) and how big the impact will be for a given resource budget (ie, the cost-effective criterion). Various stakeholders might evaluate these four criteria differently because of differing goals, priorities, and resources.

We note that resource costs and affordability are distinct; cost is the resource price tag whereas affordability is the ability to pay. For human infectious diseases, public health intervention cost-effectiveness is usually quantified in disability-adjusted life-years (DALYs) averted per US, Canadian, or Australian dollar but DALYs and cost were rarely reported for the 46 potential solutions. This omission highlights an important area for future research because for most stakeholders, and perhaps especially those interested in human health outcomes, cost-effectiveness and affordability will be the most important considerations when choosing a solution.

For some of the proposed solutions, the potential conservation and health impacts would likely be too small for most stakeholders to justify the cost. For example, invasive python control in Florida reduces predation pressures on native vertebrates and might reduce human exposure to the vector-borne Everglades virus.^97^ However, python eradication is costly and has not been achieved using existing resources, and maintaining continuous python control efforts at current intensities might not be feasible indefinitely (ie, potentially not sustainable). From a public health perspective, a cheaper and more direct public health or medical intervention might be preferred to python control. However, local stakeholders in Florida might value the small human health co-benefits from python control, even if their main goal is a potentially large conservation impact. As this example illustrates, sometimes a small impact in one sector (health or conservation) can be valued because it accompanies a large impact in another sector or fully addresses a local problem.

Quantifying the net value associated with all positive impacts in all sectors for a given intervention is difficult. However, identifying these potential impacts explicitly can help stakeholders to compare multisector interventions.^16^, ^21^, ^22^ Ultimately, for any given cross-sector problem, collaborations among stakeholders, economists, social scientists, and implementation scientists might be needed to determine which solution is optimal.

## Evidence-based management under uncertainty

Data-limited solutions that appear safe and feasible could be ideal for immediate research and adaptive implementation. However, strict adaptive implementation requires that multiple interventions are implemented simultaneously and compared, and approaches are subsequently modified according to what works best.^98^ This approach is often infeasible in conservation and public health programmes,^98^ and might be even more difficult for multisector solutions, leaving many data gaps unaddressed. When adaptive implementation is not possible, there might be other ways to fill in data gaps using safe implementation, such as by comparing different programmes that monitor, evaluate, and share outcomes. For example, multiple programmes are improving hygiene or health care for people who work or tour in great ape conservation areas, which could increase human health and reduce pathogen spillover from humans to apes.^99^ Comparing outcomes across these programmes might provide new insights for the Best Practice Guidelines for Health Monitoring and Disease Control in Great Ape Populations created by the IUCN.^100^ As evidence accumulates for this solution and others, uncertainty will decline. Data gaps are still likely to remain prominent in the near future, but action despite uncertainty will already be familiar for most public health and conservation practitioners.^9^, ^10^

## Conclusions

The growing Planetary Health field emphasises the links between human wellbeing and ecosystem integrity, but there has been scarce guidance for how to leverage these relationships to implement viable win–win solutions that specifically reduce human infectious disease burden. Here, we identified 46 such potential solutions. We found that proposed solutions address diverse, context-dependent, and dynamic threats with cross-sector interventions that are equally diverse. Numerous solutions had the potential to be safe and feasible under predictable contexts and some were supported by medium and high quality evidence of success. Some solutions had the potential for large human health or conservation impacts, such as forest conservation projects and health system strengthening initiatives. Others had small effects but might still be highly valued by local stakeholders. Synergies such as these might be pivotal for achieving the soon-to-be-revised Sustainable Development Goals.^16^, ^21^, ^22^, ^101^

Although promising, all the proposed solutions had some evidence gaps and, collectively, they did not cover all possible health and conservation threats. Evidence regarding conservation and health impacts (quantitative outcomes) and intervention cost-effectiveness and affordability were especially scarce, highlighting priorities for future research. Currently, these data gaps within and among solutions complicate decision making. More evidence will accumulate when stakeholders invest in research, adaptive implementation, monitoring, and evaluation for existing approaches. New solutions will also be created, filling in existing gaps or addressing new problems. Until then, the viability criteria described here can be used to compare and update the evidence database for potential solutions, differentiating the solutions that do not work from those that successfully and cost-effectively advance health and conservation.

### Search strategy and selection criteria

To find and synthesise evidence among proposed solutions, we used a subject-wide evidence synthesis, a two-phase approach for identifying and assessing a broad suite of interventions supported by heterogeneous evidence (Shackelford and colleagues, 2019; Sutherland and colleagues, 2018). In the first phase, we performed a systematic literature review of peer-reviewed papers and book chapters, which we used to identify solutions that have been proposed to reduce human infectious disease burdens and advance conservation goals (appendix pp 2–4). In the second phase, we performed targeted rapid reviews of peer-reviewed and grey literature (Grant and Booth, 2009), iteratively revising evidence summaries for each proposed solution (appendix pp 4–5). Finally, we used these evidence summaries to categorise information for each solution, making it easier to synthesise and compare.

To create a list of proposed win–win solutions (phase 1), we systematically reviewed publications in Thomson Reuters Web of Science and PubMed (n=12 270 papers), including records published between database inception and March 14, 2018. We performed the search using 167 English terms regarding conservation, ecology, infectious disease, and human populations (adapted from McKinnon and colleagues, 2016; appendix pp 2–4, 14). We identified 617 papers containing hypothesised or measured outcomes for conservation and human infectious diseases—excluding papers that did not discuss proposed outcomes for one or both sectors—by using a combination of researcher classification and machine learning to sort records by relevance (Cheng and colleagues, 2018). During subsequent full-text analysis, we removed any records that did not suggest at least one proposed win–win solution (eg, papers about trade-offs in which environmental degradation improves health). We then used full-text analysis of the final list of 383 records to group those pertaining to the same win–win solutions into collective case studies (appendix p 4), which resulted in a list of 46 proposed solutions.

Each solution was then individually reviewed by one or two investigators (phase 2: targeted rapid reviews), who used keyword searches to find additional peer-reviewed publications and grey literature. These rapid reviews were not systematic because they did not examine all published literature—a task that would not be possible for 46 interventions. Instead, investigators specifically sought publications relevant to 20 information categories, determined a priori, and summarised all information in a standardised format (appendix pp 4–5). A single lead investigator reviewed all collective case study summaries to ensure consistency and then each summary was reviewed by an external expert (appendix p 5). Based on feedback from external experts, investigators iteratively searched for more information and revised the collective case study summary until the investigator and lead investigator deemed the review complete.

After finishing the collective case study summary, each investigator used a list of qualitative variables defined a priori to categorise information consistently for comparison across case studies. Variables included geographical location, conservation threat, infectious disease threat, mechanism or lever type, evidence for conservation and human infectious disease outcomes, and 11 criteria that we identified as indicative of viable solutions (harmless, contained, consistent, feasible, acceptable, impactful, effective, affordable, scalable, sustainable, and cost-effective; appendix pp 7–8). The investigators’ designations were confirmed by the lead and one or two other investigators to ensure consistency. Any discrepancies between how different people categorised information were discussed until consensus was reached. Finally, we synthesised information across the 46 solutions to describe their diversity and evidence gaps.

## Data sharing

The 46 evidence summaries are publicly available in a searchable database.

## Declaration of interests

We declare no competing interests.
