# Supplementary material for: Evidence gaps and diversity among potential win–win solutions for conservation and human infectious disease control
Source: Lancet Planet Health. 2022 Aug 3;6(8):e694–705. doi: 10.1016/S2542-5196(22)00148-6 (PMC9364143; doi:10.1016/S2542-5196(22)00148-6)
Supplement: Supplementary appendix [file mmc1.pdf]

THE LANCET
Planetary Health

## Supplementary appendix

This appendix formed part of the original submission and has been peer reviewed. We post it as supplied by the authors.

Supplement to: Hopkins SR, Lafferty KD, Wood CL, et al. Evidence gaps and diversity among potential win–win solutions for conservation and human infectious disease control. *Lancet Planet Health* 2022; **6:** e694–705.

## Appendix for "Diversity and evidence gaps among potential win–win solutions for conservation and human infectious disease control"

**Authors:** Skylar R. Hopkins, Kevin D. Lafferty, Chelsea L. Wood, Sarah H. Olson, Julia C. Buck, Giulio A. De Leo, Kathryn J. Fiorella, Johanna L. Fornberg, Andres Garchitorena, Isabel J. Jones, Armand M. Kuris, Laura H. Kwong, Christopher LeBoa, Ariel E. Leon, Andrea J. Lund, Andrew J. MacDonald, Daniel C. G. Metz, Nicole Nova, Alison J. Peel, Justin V. Remais, Tara E. Stewart Merrill, Maya Wilson, Matthew H. Bonds, Andrew P. Dobson, David Lopez Carr, Meghan E. Howard, Lisa Mandle, Susanne H. Sokolow

### General overview of our subject-wide evidence synthesis approach:

In this project, we collected, evaluated, and synthesized information about 46 proposed win–win solutions using a method called a "subject-wide evidence synthesis" (Fig. 1). This method was designed for evidence synthesis in situations where (i) there is no *a priori* list of interventions to review (the intervention list is generated by the review); (ii) there are too many interventions to review each using a systematic literature review (because doing so would be prohibitively costly); and (iii) the evidence among interventions is too limited or variable to perform a meta-analysis for each intervention.[1] The subject-wide evidence synthesis method first uses a classic systematic literature review to identify a landscape of possible interventions (Phase 1, Section 1 below), and then evidence for each intervention is assessed using a secondary targeted review process called a "rapid review" (Phase 2, Section 2 below).[1] This two-phase method sacrifices some of the quantitative rigor from a classic systematic literature review and meta-analyses of a few interventions in order to identify and assess a broader suite of interventions supported by evidence of variable quality and quantity.[1] In this section of the Supplementary Methods, we generally describe the two phases as they applied to our synthesis of proposed win–win solutions, and then we explain each phase in more detail below.

The first step in our subject-wide evidence synthesis was to use a systematic literature review to create a list of win–win solutions proposed to both reduce human infectious disease burdens and advance conservation goals (Fig. 1, Section 1 below). In a preliminary scoping review, we discovered that many relevant, peer-reviewed papers would not include terms like "win–win solution", "intervention", "synergy", or "co-benefit". Instead of relying on these terms to find relevant literature, we performed a broader systematic literature review of peer-reviewed papers with terms related to conservation, ecology, infectious diseases, and humans. This search method was more inclusive but also returned many papers that did not hypothesize outcomes for conservation and/or human infectious diseases. To more efficiently sort the relevant from irrelevant papers, we used the colandr machine learning algorithm, which learns from researchers' decisions to include or exclude papers and sorts the remaining papers by relevance.[2] Using this combination of researcher classification and machine learning sorting, we filtered the initial list of 12,270 papers to a list of 617 papers containing proposed or measured outcomes for both

conservation and human infectious diseases (see PRISMA diagram in Supplemental Fig. 1). Many of these papers were about the same few solutions, and many more did not include proposed win–win solutions (e.g., papers that were about trade-offs where human health was improved through environmental degradation). Therefore, we used full-text analysis to again exclude irrelevant papers. We then group papers that involved similar "levers" or mechanisms into collective case studies. This created a final list of 46 unique proposed win–win solutions to evaluate with targeted reviews (Table 1).

The second step in our subject-wide evidence synthesis was to perform a rapid review of each intervention and summarize the resulting information (Fig. 1, see Section 2 below). We began each rapid review with a list of 20 qualitative categories of information that we wanted to summarize (e.g., collateral impacts caused by the intervention on the environment, social acceptability of the intervention), and we iteratively searched the peer-reviewed and gray literature for information relevant to these categories. As one or two investigators performed these reviews and summarized the resulting information, a single lead investigator (SRH) iteratively reviewed each summary to ensure that all rapid reviews were being performed in a consistent way across investigators. When the investigators and lead investigator were satisfied with the collective case study summary, it was sent to at least one expert for a friendly peer review. Based on the expert's feedback, the summary was again revised by the investigator and double-checked again by the lead investigator. Finally, the information contained within each summary was evaluated and classified in several ways (e.g., identifying the primary conservation threat) so that we could synthesize this information across proposed solutions.

**Systematic literature review to create a list of proposed win–win solutions**

Our systematic literature review was based on five types of search terms: conservation terms, ecological qualifier terms, infectious disease terms, human qualifier terms, and exclusion terms (see each list below). This methodology generally followed a previous evidence mapping study conducted by McKinnon et al.,[3] but we modified that study's search terms to focus specifically on outcomes related to human infectious diseases and to include some additional terms that we deemed important based on an initial scoping review of relevant papers. In particular, the conservation terms were compiled from McKinnon et al.[3] and the list of conservation threats identified by the International Union for the Conservation of Nature,[4] plus two terms that were frequently and prominently used in highly-relevant papers that we found in an initial scoping review of the literature: "environmental change" and preserv*. The ecological qualifier terms came from McKinnon et al.[3] and a list of biomes,[5] plus the term "ecosystem function", which we found in several relevant papers in an initial scoping review. The human qualifiers came from McKinnon et al.,[3] but we did not include some of their terms that returned results that were not relevant to our search (i.e., community, fisher, collaborator), and we added terms related to children/childhood. Finally, the infectious disease terms came from lists maintained by the National Institute of Allergy and Infectious Disease[6] and the World Health Organization,[7] plus three terms we deemed relevant

based on an initial scoping review: “disease control”, “disease risk”, and “infection risk”. We also excluded any papers about “oxygen species”, which were abundant and not relevant to our study. The final search string took the form of ("conservation qualifier term 1" OR "conservation qualifier term 2") AND ("ecology term 1" OR "ecology term 2") AND ("infectious disease term 1" OR "infectious disease term 2") AND ("human qualifier term 1" OR "human qualifier term 2") NOT (“oxygen species”).

CONSERVATION TERMS: ("capacity-building" OR "invasive species" OR “climate change” OR “ecosystem service*” OR “fire suppression” OR “population decline*” OR “re-introduction” OR agriculture OR aquaculture OR conserv* OR dam OR ecotourism OR effluent OR “environmental change*” OR extinction* OR extirpation OR farm* OR fishing OR harvesting OR hunting OR law* OR logging OR management OR mining OR outreach OR policy* OR pollution OR preserv* OR reintroduction OR reservation OR stewardship OR sustainab* OR transportation OR war)

ECOLOGICAL QUALIFIER TERMS: AND (marine OR freshwater OR coastal OR forest* OR ecosystem* OR species OR habitat* OR biodiversity OR ecolog* OR landscape OR seascape OR “coral reef*” OR “natural resource*” OR grassland OR savanna OR desert OR taiga OR tundra OR wetland OR aquatic OR estuary OR river OR stream OR lake OR pond OR jungle OR “ecosystem function”)

HUMAN QUALIFIER TERMS: AND (human* OR childhood OR children OR household OR people)

INFECTIOUS DISEASE TERMS: AND (Tuberculosis OR Mycobacterium OR “sexually transmitted disease*” OR Syphilis OR Chlamydia OR Gonorrhoea OR Trichomoniasis OR Herpes OR Herpesvirus OR “Haemophilus ducreyi” OR “Klebsiella granulomatis” OR Papillomavirus OR HIV OR AIDS OR Diarrhoeal OR diarrhea* OR Shigella OR Salmonella OR Cholera OR “Escherichia coli” OR “Yersinia enterocolitica” OR Campylobacter OR “Clostridium difficile” OR “Entamoeba histolytica” OR “Balantidium coli” OR Giardia OR Cryptosporidium OR Isospora OR Sarcocystis OR Rotavirus OR Adenovirus OR “Norwalk agent” OR “Whooping cough” OR Pertussis OR Diphtheria OR Measles OR Tetanus OR Meningitis OR Streptococcus OR “Naegleria fowleri” OR Encephalitis OR “Murray Valley virus” OR “Kunjin virus” OR “La Crosse virus” OR “Rocio virus” OR “Louping ill virus” OR “Powassan virus” OR Hepatitis OR Malaria OR Plasmodium OR trypanosom* OR “Chagas disease” OR schistosom* OR Leishmania* OR “Lymphatic filariasis” OR Onchocerciasis OR Cysticercosis OR Echinococcus OR Dengue OR Trachoma OR “Yellow fever” OR Rabies OR Nematode OR Ascariasis OR Trichuriasis OR Hookworm OR Food-borne OR Foodborne OR Food-bourne OR Trematod* OR Strongyloid* OR Enterobiasis OR Anisakiasis OR “Intestinal capillariasis” OR Trichostrongyliasis OR Leprosy OR Zoonosis OR Zoonotic OR Whipworm

OR Chikungunya OR Ehrlichiosis OR Influenza OR Leptospirosis OR “Lyme disease” OR Norovirus OR Plague OR Scabies OR Rotavirus OR Toxocar* OR “West nile virus” OR Toxoplasm* OR “Sin nombre virus” OR “Hanta virus” OR “Zika virus” OR Ebola OR Drucunculiasis OR “Infectious disease” OR Arbovirus OR Vectorborne OR Parasit* OR “Disease control”).

We used these English search terms to query two databases: Thomson Reuters Web of Science and NCBI PubMed. We put the keyword string within Web of Science’s “topic” field (includes title, abstract, and keywords) and within PubMed’s “title/abstract” field. Both searches were completed on 14 March 2018 and returned 10,094 and 3,723 references, respectively (Supplementary Fig. 1). There were no restrictions on the publication dates for included records. We downloaded the results as .bib and .nbib files, used Zotero to consolidate references, and uploaded all results to colandr (see below). After colandr removed duplicates, we were left with 12,270 references (Supplementary Fig. 1).

We used colandr[2]—an open access, machine-learning assisted literature review platform—to review the titles and abstracts of all records and classify those that hypothesized or measured outcomes for both human infectious disease and conservation (Supplementary Fig. 1). Each title and abstract were read by two people who independently determined whether the paper did or did not hypothesize or measure outcomes for both sectors. Any papers that were scored differently (include vs. exclude) by the two readers were discussed until a conservative consensus was reached. As papers were classified to be included vs. excluded, the machine-learning algorithm sorted the remaining papers by relevance based on keywords, such that readers were prioritizing screening the most relevant papers first. After the first 500 records were screened, we continued to find relevant papers, but few new proposed solutions were found (i.e., the papers were about solutions that we had already encountered at least once). After screening the 1,000th paper, no new solutions were found as we continued screening through thousands more abstracts. Therefore, we assumed that all proposed solutions in the literature had been found by the time we reached the 4,940th paper, and we excluded all records that had been sorted as less relevant by colandr and had not been screened by that point (7,330 records; Supplementary Fig. 1). When we finished screening records based on whether they included hypothesized or measured outcomes for both conservation and human infectious diseases, we had a list of 617 records to include in our full-text analysis (Supplementary Fig. 1).

During full-text analysis, we found that many (n=234) of the 617 papers did not include proposed win–win solutions, where we define win–win solutions as levers that act on positive correlations between human health and conservation to restore degraded systems (Fig. 1).[8–10] For example, in Australia, deforestation of flowering eucalypts caused bats to reduce nomadic movements among forests and roost near anthropogenic food sources in cities and pastures. New bat distributions increased bat–human conflicts, including increasing Hendra virus spillover from bats to horses and

then to humans[11]—a lose–lose scenario. We removed all such lose–lose scenarios during our full-text analysis unless at least one paper also suggested a relevant solution. For example, to improve bat and human health, a landscape-level revegetation project has been initiated to support nomadic bat movements, which is expected to reduce spillover risk—a win–win solution. Unlike this lose–lose to win–win trajectory, other papers focused on preventing degradation by maintaining baseline conditions, and those solutions would be better classified as win–neutral or neutral–neutral solutions.[10] For example, many papers focused on conserving intact forests (i.e., no change in forest cover), because preventing deforestation is expected to prevent contacts and spillover between wildlife and people.[12–16] In our review, we retained win–neutral or neutral–neutral solutions that maintained healthy baselines and win–win solutions that reduced human infectious disease burdens while improving ecosystem structure or function. In all these cases included in our final list of 46 solutions, health and conservation were proposed to be better with the intervention than without it.

Some of the 234 papers that we removed during our full-text analysis were generally about improving both conservation and reducing infectious disease burdens, but the underlying mechanisms were too diffuse or poorly articulated for us to cover in a subsequent review. These covered five important topics that should still be considered by future research and management in these sectors: (i) interventions to mitigate global climate change, which exacerbates both ecosystem degradation and human infectious diseases at local and global scales;[17,18] (ii) interventions to prevent forest/habitat fragmentation separately from habitat loss/degradation, because fragmentation and edge habitats have been linked to zoonotic spillover;[19,20] (iii) efforts to improve interdisciplinary collaboration through the relatively new One Health, EcoHealth, and Planetary Health fields;[21,22] (iv) efforts to improve surveillance of wildlife and/or people to detect disease outbreaks earlier;[23,24] and (v) selective breeding or genetic modification of wildlife or livestock to reduce susceptibility to diseases shared by wildlife, livestock, and people.[25,26] After excluding papers about these diffusely synergistic examples and the papers that did not include at least one proposed win–win solution, we were left with 383 papers (Supplementary Fig. 1).

Finally, we used our full-text analysis to turn the list of 383 papers containing proposed win–win solutions into a list of 46 unique proposed solutions (Supplementary Fig. 1). In particular, we combined papers or examples where the underlying "lever" or mechanism creating the conservation outcomes and human infectious disease outcomes were the same into collective case studies. For example, there were many papers about the dilution effect, a proposed mechanism whereby restoring or conserving wildlife biodiversity can reduce human disease risk.[27] Most of these papers were specific to Lyme disease, but several other papers considered the same underlying dilution effect mechanism in other disease systems—"diluting" sylvatic transmission from the most competent wildlife host species. These were all combined in one collective case study on the general and Lyme-specific dilution effect. There were also a few papers regarding the dilution effect and Chagas disease, which relied on a slightly different lever (most Chagas

transmission is peridomestic, complicating the relationship between wildlife biodiversity, sylvatic transmission, and human risk), so we created a second, separate collective case study for studies related to the Chagas-specific dilution effect. Using our collective expert opinions, we continued combining papers in this way until all 46 unique proposed solutions were listed.

**Rapid reviews and collective case study summaries**

To develop a standardized method for reviewing and summarizing collective case studies, a team of four individuals wrote and iteratively revised one-page summaries for two collective case studies that focused on research from members of the working group: one on restoring prawns to reduce schistosomiasis and one on restoring/conserving forests to reduce childhood diarrhea. Based on this process, the team iteratively developed a standardized list of important informational categories that could be used to examine all collective case studies in the same critical, structured way, and this list was later critiqued and revised by 15 people at an in-person group meeting. The final list of 20 categories included in these one page summaries were as follows: human infectious disease (HID), conservation problem, intervention, target (of the intervention), mechanism (by which the intervention works), HID evidence, conservation evidence, location and spatial scale, temporal scale, health success metrics, conservation success metrics, human co-benefits, conservation co-benefits, collateral human impacts, collateral conservation impacts, social acceptability, equity considerations, practicality or challenges, status, research needs/gaps. Definitions of each of these categories were described in an instruction document (also see below). The instruction document also defined eight types of evidence (see below), established a one-page (single-spaced) limit for summaries, and noted that because cost-effectiveness or economic studies were anticipated to be rare, any such studies should be explicitly noted in the collective case study summaries. This instruction document and the two example one-page collective case study summaries were provided to all collective case study investigators.

After the final list of 46 collective case studies and the instruction document were completed, each collective case study was assigned to one summary investigator (or occasionally a team of two investigators). Investigators then conducted a targeted literature review of published peer-reviewed papers (using search terms that they deemed relevant) and Internet-searchable gray literature to find all relevant information in the 20 categories listed above. There were no restrictions on the publication dates for included records. Searches for peer-reviewed literature relied on Web of Science and Google searches, and investigators also examined the reference lists of relevant papers to find other peer reviewed literature. Investigators used this information to structure and write a one-page summary, iteratively returning to searching until all 20 information categories had been sufficiently covered. In the absence of literature, investigators were encouraged to use their expert opinion and knowledge of the system to suggest possible outcomes for categories such as equity considerations or collateral impacts, but to be clear that literature was missing for those categories. For instance, forest conservation has historically involved disenfranchising local people, so all summaries related to this topic note this *potential* equity concern, even if the literature for the

collective case study did not discuss equity. Alternatively, when information was missing or a category was not relevant to the case study, acceptable responses included “unknown” or “not applicable”. If different sources provided contrary information or evidence, investigators described all reported evidence, and highlighted the sentences including the contradictory evidence in yellow, such that all future readers would note the contradiction.

Roughly 25% of each one-page summary was devoted to detailed descriptions of the evidence available to support the proposed HID outcomes and conservation outcomes from the intervention. Authors used uppercase designations before sentences in these evidence sections to specify the type of evidence being reported: ANECDOTE, HYPOTHESIS, OBSERVATIONAL STUDY (e.g., no experimental manipulation or treatment applied), BEFORE-AFTER STUDY (comparing pre- to post- intervention or natural experiment outcomes, usually for one or a few sites), EXPERIMENT (where applicable, randomized control trials were designated as their own category, separate from experiments), THEORETICAL MODEL, SYSTEMATIC LITERATURE REVIEW, META-ANALYSIS, and REVIEW. At least one secondary group member reviewed each evidence section to be sure that no evidence was missing, especially by checking for any relevant conservation evidence types on conservationevidence.com and by looking for any published systematic literature reviews on the Cochrane Database of Systematic Reviews. Any additional studies found on these websites were added to the one-page summary. The reference section, which was separate from the one-page summary, was then organized by the evidence categories. Within this organization scheme, a single reference could be placed under multiple categories, and helpful but non-critical references were placed under a SUPPORTING INFORMATION category.

Once drafted, the one-page summary was iteratively revised based on feedback from a single lead investigator (SRH), who ensured that all summaries were written in a consistent way, following the standardized format. The summary was then informally reviewed by at least one external researcher or practitioner familiar with the intervention and/or socio-ecological system described by the collective case study. Friendly reviews were solicited over email, where we specifically contacted authors of recent, relevant, peer-reviewed literature or practitioners involved in intervention implementation. In the email, we requested that friendly reviewers not only check the summaries for accuracy, but also to help us identify any crucial gaps in our case studies, especially missing collateral impacts or logistical challenges and related ongoing projects that were not yet published. Specific comments and general feedback from friendly reviewers were then incorporated into the one-page summaries.

After the literature review, writing, peer-review, and revision were completed—a process that took approximately 40 person-hours per collective case study—the final summaries were all included on a public-facing website: https://ecohealthsolutions.stanford.edu/research/win-win-solutions-people-and-nature

**Evaluation and synthesis: categorizing information from the rapid reviews**

To synthesize knowledge across the 46 proposed win–win solutions, we first needed to categorize information from each collective case study in a way that was comparable across the diverse solutions. In particular, we wanted to be able to describe diversity and evidence gaps among solutions with regards to their geographic distributions, the conservation threats they addressed, the human infectious diseases they addressed, the type of "lever" or mechanism used to achieve the conservation and health outcomes, the type and strength of evidence available to support the proposed conservation and health outcomes, and 11 criteria that we identified as indicative of "viable" solutions. Below we describe how we consistently categorized each literature summary according to these themes. The full dataset resulting from these categorizations is available on the open access website containing the collective case study summaries.

*Geographical Information:*

Because our collective case study summaries typically incorporated several case studies (e.g., several different community conservation programs in several different African countries have incorporated HIV/AIDS initiatives), few of the collective case studies could be assigned to specific geographical coordinates. Instead, we classified the extent of each collective case study in a categorical way, using designations for multi-continent (global), multi-country within a continent, or single country solutions. We then chose a single, generalized geographical location to represent the collective case study on a map (Figure 3A). For country-specific collective case studies, this point was placed in the middle of the country or in the most relevant town/village/national park. For multi-country and multi-continent collective case studies, this point was placed in a single country where a particularly notable case study occurred. As a result, the map in Figure 3A does not show every location where a collective case study could be applied, but rather the conservative diversity of locations where the relevant interventions/studies have been applied.

*Conservation Threats:*

The IUCN categorizes 12 types of conservation threats: residential & commercial development; agriculture & aquaculture; energy production & mining; transportation & service corridors; biological resource use; human intrusions & disturbance; natural system modification; invasive & other problematic species, genes & diseases; pollution; geological events; climate change & severe weather; or other.[4] We used this list and the IUCN's definitions of each to determine which threats were relevant to each collective case study, as well as which threat was of primary relevance to the collective case study.

*Pathogen Type:*

Humans have had documented infections from more than 1,400 known parasite or pathogen species,[13] which belong to many disparate taxonomic clades and nonliving groups (viruses and prions). To better understand which parts of this parasite biodiversity were and were not captured

by existing win–win solutions, we recorded whether each collective case study involved the following major taxonomic groups: prions (infectious proteins), viruses, bacteria, protozoa, fungi, arthropods, and helminths. Collective case studies often involved more than one human infectious disease, and thus they could also involve more than one major parasite group. Even when multiple pathogens or parasites were involved, we could usually identify one major transmission pathway involved in each collective case study (direct contact, waterborne, vector-borne, zoonotic from livestock, or zoonotic from wildlife), so we also recorded this major transmission pathway.

*Lever Type:*

As we reviewed the proposed solutions, we found that many involved one of the seven IUCN conservation action classes: land/water protection; land/water management; species management; education & awareness; law & policy; livelihoods, economic & other incentives; or external capacity building. Using the IUCN's definitions of these action classes, we designated which was most relevant to each solution. However, it seemed inappropriate to call many of the solutions "conservation levers", which implies that a conservation intervention primarily acts on ecosystems and then has some secondary or simultaneous benefit for human health. Instead, many of the solutions were public health interventions (e.g., vector control) that had some secondary or simultaneous benefit for conservation (i.e., a health lever for conservation). We therefore also categorized the solutions by lever type to summarize how many were primarily conservation levers for health, health levers for conservation, or levers for health and conservation (interventions not specific to health or conservation but beneficial to both in this context).

*Evidence Quality:*

There was notable variation among the proposed solutions in the types of evidence available to support their proposed outcomes: some had never been the focus of a primary peer-reviewed study (i.e., they had only been hypothesized to work in a review or perspective paper), some had been studied frequently enough that they had previously been the focus of a meta-analysis, some were supported by the same consistent outcomes in all studies, some had contradictory outcomes across studies, etc. To describe this variation, we evaluated the quality of evidence for the proposed outcomes in each collective case study using an evidence rubric that we modified from a rubric used by the Bridge Collaborative (Table S1).[28] Using this three-part rubric, authors assigned a 0 to 3 rating regarding the evidence diversity, evidence consistency (also see Viability Criteria below), and evidence applicability. The scores for each of these categories were then combined into a metric of overall evidence confidence, where the overall score was the minimum score for any given category (e.g., 3, 1, 2 would receive an overall score of 1, or "low"). After the investigators of each summary had evaluated the evidence using this rubric, their evaluation was checked by two other investigators. Whenever independent evaluations differed, they were discussed until consensus was achieved. Evidence evaluation for the proposed human infectious disease outcomes and conservation outcomes was done separately, such that each collective case study could be placed on the two-dimensional matrix in Figure 3.

*Viability Criteria:*
During the evaluation process, it became clear that most collective case studies had major data gaps among the 20 information categories that we reviewed and/or the proposed solution had some potential or documented negative impacts on socio-ecological systems that could make the solution unacceptable for some stakeholders. We discussed these issues at in-person working group meetings and via subsequent online discussions, through which we defined and iteratively revised a set of 11 criteria for viable solutions that one could potentially assess across all solutions: Harmless, Contained, Consistent, Feasible, Acceptable, Impactful, Effective, Affordable, Scalable, Sustainable, and Cost-effective (Fig. 4). Below we define each of the criteria. For five criteria (Harmless, Contained, Consistent, Feasible, Acceptable), the collective case study investigator used the information from the review to evaluate whether the criterion was met (Yes) or unmet (No), or whether there was not enough information to evaluate with certainty (Data Deficient). If needed, the investigator completed additional literature reviews or updated the one-page summary to clarify this information. Two additional investigators then independently checked these evaluations for all 46 collective case studies to ensure consistent decision-making. Whenever independent evaluations differed, they were discussed until consensus was achieved. We did not evaluate the other six criteria (Impactful, Effective, Affordable, Scalable, Sustainable, and Cost-effective), because evaluation would be context-dependent for different individuals/organizations intent upon funding, researching, or implementing each intervention.

Harmless (i.e., no negative externalities for people): The intervention does not notably disenfranchise the poor or people with limited political or social power. Note that an intervention that benefits some people more than others can still be harmless under this definition, as long as the intervention does not benefit some people at the expense of other people.

Contained (i.e., no negative externalities for natural systems): The intervention has no notable collateral impacts for ecosystem structure or function, or if potential collateral impacts exist, there are known methods to mediate or avoid them.

Consistent: The intervention has not had or is not likely to have the opposite effect on the human infectious disease target (e.g., increase instead of decrease burdens) or the conservation target (e.g., reduce ecosystem integrity instead of increase ecosystem integrity). Note that evidence showing no effect on a target does not qualify as inconsistency; the effect must be in the opposite direction from that desired to say it is not consistent. Interventions that are not consistent should not be implemented unless very specific scenarios where consistency is assured can be found.

Feasible: The intervention could be successful if implemented today, given existing evidence, structural and logistical capacity, and technology.

Acceptable: The intervention is already socially acceptable or could be easily made acceptable by making modifications to appease particular stakeholder groups.

Impactful: The intervention has a large enough impact on human health or conservation that it is worth doing. Individuals or organizations will evaluate this criterion differently for any collective case study depending on their specific goals.

Effective: The intervention has worked, or it is likely enough to work that it is worth doing. Individuals or organizations will evaluate this criterion differently for any collective case study depending on their willingness to invest in an intervention that might have no effect.

Affordable: The intervention could be paid for in full by the people tasked to pay for the intervention. Individuals or organizations might evaluate this criterion differently for any collective case study depending on their budgets.

Scalable: The intervention has been or could be scaled up to cover most/all of the spatial extent of the problem. Individuals or organizations might evaluate this criterion differently for any collective case study depending on their specific definitions of the extent of the problem (e.g., national organization covering extent in country vs. international organization covering global extent).

Sustainable: The intervention has been or could be implemented long enough to achieve human health and conservation goals. Individuals or organizations might evaluate this criterion differently for any collective case study depending on their specific goals (e.g., reduce disease burdens 50% vs. eradicate disease).

Cost-effective: The intervention has a big enough impact to justify the cost. Individuals or organizations might evaluate this criterion differently for any collective case study depending on their goals and budgets.

**References for Supplementary Methods:**

1 Sutherland WJ, Wordley CFR. A fresh approach to evidence synthesis. *Nature* 2018; **558**: 364–6.

2 Cheng SH, Augustin C, Bethel A, *et al.* Using machine learning to advance synthesis and use of conservation and environmental evidence. *Conserv Biol* 2018; **32**: 762–4.

3 McKinnon. What are the impacts of nature conservation interventions on human well-being: A systematic map protocol. *Environ Evid* 2016. https://www.researchgate.net/publication/264488396_What_are_the_impacts_of_nature_conservation_interventions_on_human_well-being_A_systematic_map_protocol (accessed Nov 13, 2018).

4 Threats Classification Scheme (Version 3.2). IUCN Red List Threat. Species. https://www.iucnredlist.org/en (accessed April 11, 2021).

5 The world's biomes. UC Mus. Paleontol. https://ucmp.berkeley.edu/exhibits/biomes/index.php (accessed April 11, 2021).

6 NIAID Emerging Infectious Diseases/Pathogens. Natl. Inst. Allergy Infect. Dis. https://www.niaid.nih.gov/research/emerging-infectious-diseases-pathogens (accessed April 11, 2021).

7 WHO. WHO methods and data sources for global burden of disease estimates 2000-2015. Geneva: Department of Information, Evidence and Research WHO, 2017.

8 International Council for Science. A Guide to SDG Interactions: from Science to Implementation. 2017 https://council.science/publications/a-guide-to-sdg-interactions-from-science-to-implementation/.

9 Allen C, Metternicht G, Wiedmann T. Prioritising SDG targets: assessing baselines, gaps and interlinkages. *Sustain Sci* 2019; **14**: 421–38.

10 Hopkins SR, Sokolow SH, Buck JC, *et al.* How to identify win–win interventions that benefit human health and conservation. *Nat Sustain* 2020; : 1–7.

11 Giles JR, Eby P, Parry H, *et al.* Environmental drivers of spatiotemporal foraging intensity in fruit bats and implications for Hendra virus ecology. *Sci Rep* 2018; **8**: 9555.

12 Karesh WB, Dobson A, Lloyd-Smith JO, *et al.* Ecology of zoonoses: natural and unnatural histories. *The Lancet* 2012; **380**: 1936–45.

13 Taylor LH, Latham SM, Woolhouse ME. Risk factors for human disease emergence. *Philos Trans R Soc Lond B Biol Sci* 2001; **356**: 983–9.

14 White RJ, Razgour O. Emerging zoonotic diseases originating in mammals: a systematic review of effects of anthropogenic land-use change. *Mammal Rev* 2020. DOI:10.1111/mam.12201.

15 Jones KE, Patel NG, Levy MA, *et al.* Global trends in emerging infectious diseases. *Nature* 2008; **451**: 990–3.

16 IPBES. Workshop Report on Biodiversity and Pandemics of the Intergovernmental Platform on Biodiversity and Ecosystem Services (IPBES). Bonn, Germany: IPBES Secretariat, 2020 DOI:10.5281/zenodo.4158500.

17 Tong S, Mather P, Fitzgerald G, McRae D, Verrall K, Walker D. Assessing the Vulnerability of Eco-Environmental Health to Climate Change. *Int J Environ Res Public Health* 2010; **7**: 546–64.

18 Pecl GT, Araújo MB, Bell JD, *et al.* Biodiversity redistribution under climate change: Impacts on ecosystems and human well-being. *Science* 2017; **355**. DOI:10.1126/science.aai9214.

19 Ducheyne E, Mweempwa C, De Pus C, *et al.* The impact of habitat fragmentation on tsetse abundance on the plateau of eastern Zambia. *Prev Vet Med* 2009; **91**: 11–8.

20 Wilkinson DA, Marshall JC, French NP, Hayman DTS. Habitat fragmentation, biodiversity loss and the risk of novel infectious disease emergence. *J R Soc Interface* 2018; **15**: 20180403.

21 Rabinowitz P, Conti L. Links among human health, animal health, and ecosystem health. *Annu Rev Public Health* 2013; **34**: 189–204.

22 Romanelli C, Cooper HD, de Souza Dias BF. The integration of biodiversity into One Health. *Rev Sci Tech Int Off Epizoot* 2014; **33**: 487–96.

23 Thanapongtharm W, Linard C, Wiriyarat W, *et al.* Spatial characterization of colonies of the flying fox bat, a carrier of Nipah Virus in Thailand. *BMC Vet Res* 2015; **11**: 81.

24 Fuller T, Bensch S, Müller I, *et al.* The ecology of emerging infectious diseases in migratory birds: an assessment of the role of climate change and priorities for future research. *EcoHealth* 2012; **9**: 80–8.

25 Adalja A, Sell TK, McGinty M, Boddie C. Genetically Modified (GM) Mosquito Use to Reduce Mosquito-Transmitted Disease in the US: A Community Opinion Survey. *PLoS Curr* 2016; **8**. DOI:10.1371/currents.outbreaks.1c39ec05a743d41ee39391ed0f2ed8d3.

26 le Roex N, Berrington CM, Hoal EG, van Helden PD. Selective breeding: the future of TB management in African buffalo? *Acta Trop* 2015; **149**: 38–44.

27 LoGiudice K, Ostfeld RS, Schmidt KA, Keesing F. The ecology of infectious disease: Effects of host diversity and community composition on Lyme disease risk. *Proc Natl Acad Sci* 2003; **100**: 567–71.

28 Tallis H, Kreis K, Olander L, *et al.* Aligning evidence generation and use across health, development, and environment. *Curr Opin Environ Sustain* 2019; **39**: 81–93.

**Supplementary Fig. 1.** This PRISMA diagram outlines the first step in our subject-wide evidence synthesis, which was a systematic literature review used to create a list of win–win solutions proposed to both reduce human infectious disease burdens and advance conservation goals. Using a combination of human classification and machine learning, we filtered a list of 12,270 peer-reviewed papers and book chapters, ending with a list of 383 records that contained at least one proposed win–win solution.

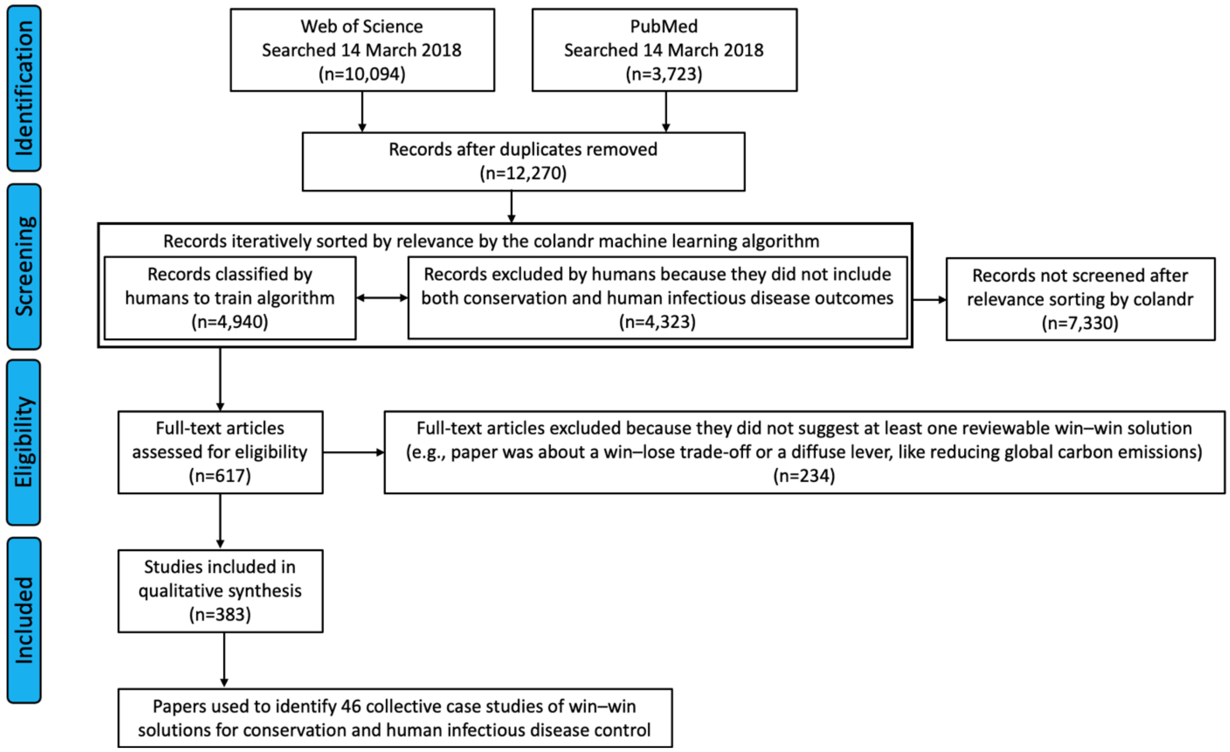

**Supplementary Table 1.** The three-part rubric used to evaluate the evidence for the proposed human infectious disease outcomes and the proposed conservation outcomes for each collective case study.

| Overall Evidence Confidence | Evidence Types and Diversity | Evidence Consistency | Evidence Applicability |
|---|---|---|---|
| High | 3: Diverse evidence, including at least one intervention study, leaves little uncertainty regarding the outcome and the underlying mechanisms. | 3: consistent | 3: evidence addresses the contexts relevant to the problem |
| Medium | 2: Several lines of evidence, including experimental manipulations, address most of the important questions and underlying mechanisms that affect the outcome of interest. | 2: mostly consistent; inconsistency easy to explain | 2: existing studies mostly occur at the geographical locations, spatial and temporal scales, or socio-ecological contexts relevant to the problem |
| Low | 1: There are a few observational studies (e.g., before-after or correlational studies) that support hypotheses or model predictions. The existing studies leave major knowledge gaps and/or the underlying mechanisms for observed outcomes are not tested. | 1: moderate to major inconsistency; inconsistency difficult to explain | 1: existing studies mostly do not occur at the geographical locations, spatial and temporal scales, or socio-ecological contexts relevant to the problem |
| None | 0: None. There are only hypotheses and/or anecdotes (e.g., in review/opinion papers). | NA | NA |

**Supplementary Table 2.** The 383 papers from the systematic literature review that contained at least one proposed win–win solution, which were used to create the final list of 46 proposed solutions.

| Title | Authors | Journal or Book | Year |
|---|---|---|---|
| Consolidation Of International Guidelines For The Management Of Canine Populations In Urban Areas And Proposal Of Performance Indicators. | Garcia et al. | *Rev Panam Salud Publica* | 2012 |
| Ecosystem Health And Human Health. | Cecchi and Mancini | *Ann 1St Super Sanita* | 2005 |
| Use Of Remote Sensing To Study The Influence Of Environmental Changes On Malaria Distribution In The Brazilian Amazon. | Vasconcelos et al. | *Cad Saude Publica* | 2006 |
| A Framework For The Integration Of Ecosystem And Human Health In Public Policy: Two Case Studies With Infectious Agents | Koren and Crawford-Brown | *Environmental Research* | 2004 |
| A Framework For The Study Of Zoonotic Disease Emergence And Its Drivers: Spillover Of Bat Pathogens As A Case Study | Wood et al. | *Philosophical Transactions Of The Royal Society B Biological Sciences* | 2012 |
| A Global Assessment Of Closed Forests, Deforestation And Malaria Risk | Guerra et al. | *Annals Of Tropical Medicine And Parasitology* | 2006 |
| A Long-Term Monitoring Study Of Chlorophyll, Microbial Contaminants, And Pesticides In A Coastal Residential Stormwater Pond And Its Adjacent Tidal Creek | DeLorenzo et al. | *Environmental Monitoring And Assessment* | 2012 |
| A Meta-Analysis Suggesting That The Relationship Between Biodiversity And Risk Of Zoonotic Pathogen Transmission Is Idiosyncratic | Salkeld et al. | *Ecology Letters* | 2013 |
| A One Health Message About Bats Increases Intentions To Follow Public Health Guidance On Bat Rabies | Lu et al. | *Plos One* | 2016 |
| A Research Framework For Identifying Potential Linkages Between Isolated Wetlands And Disease Ecology | Kirkman et al. | *Ecological Research* | 2011 |
| A Review Of Biodiversity Outcomes From Possum-Focused Pest Control In New Zealand | Byrom et al. | *Wildlife Research* | 2016 |
| A Review Of The Global Conservation Status Of Bats | Mickleburgh et al. | *Oryx* | 2002 |
| A Review Of The Interactions Between Free-Roaming Domestic Dogs And Wildlife | Hughs and Macdonald | *Biological Conservation* | 2013 |
| A Review Of Zoonotic Infection Risks Associated With The Wild Meat Trade In Malaysia | Cantlay et al. | *Ecohealth* | 2017 |
| A Study On Use Of Animals As Traditional Medicine By Sukuma Tribe Of Busega District In North-Western Tanzania | Vats and Thomas | *Journal Of Ethnobiology And Ethnomedicine* | 2015 |

| | | | |
|---|---|---|---|
| Abandonment, Ecological Assembly And Public Health Risks In Counter-Urbanizing Cities | Gulachenski et al. | *Sustainability* | 2016 |
| Abundance And Distribution Of Sylvatic Dengue Virus Vectors In Three Different Land Cover Types In Sarawak, Malaysian Borneo | Young et al. | *Parasites & Vectors* | 2017 |
| Adult Mortality And Natural Resource Use In Rural South Africa: Evidence From The Agincourt Health And Demographic Surveillance Site | Hunter et al. | *Society & Natural Resources* | 2011 |
| Advances In Aquaculture Vaccines Against Fish Pathogens: Global Status And Current Trends | Dadar et al. | *Reviews In Fisheries Science & Aquaculture* | 2017 |
| All Health Is Local: Biodiversity, Ethics, And Human Health | Butkus | *Ethics Policy & Environment* | 2015 |
| An Appraisal Of Bird-Mediated Ecological Functions In A Changing World | Morante-Filho and Faria | *Tropical Conservation Science* | 2017 |
| An Ecological Risk Assessment For Insecticides Used In Adult Mosquito Management | Davis et al. | *Integrated Environmental Assessment And Management* | 2007 |
| An Ethnomedicinal Study Of The Seri People; A Group Of Hunter-Gatherers And Fishers Native To The Sonoran Desert | Narchi et al. | *Journal Of Ethnobiology And Ethnomedicine* | 2015 |
| An Ethnozoological Study In The Adjoining Areas Of Mount Abu Wildlife Sanctuary, India. | Jaroli et al. | *J Ethnobiol Ethnomed* | 2010 |
| Animal And Human Health Implications Of Avian Influenza Infections | Capua and Alexander | *Bioscience Reports* | 2007 |
| Animal Origins Of Sars Coronavirus: Possible Links With The International Trade In Small Carnivores | Bell et al. | *Philosophical Transactions Of The Royal Society Of London Series B-Biological Sciences* | 2004 |
| Anthrax Kills Wild Chimpanzees In A Tropical Rainforest | Leendertz et al. | *Nature* | 2004 |
| Anthropogenic Environmental Change, Mosquito-Borne Diseases And Human Health In New Zealand | Derraik and Slaney | *Ecohealth* | 2007 |
| Antimicrobial Resistance To 14 Antimicrobials In Marine Coastal Waters Around Northern Ireland: Use Of The Novel Relative Resistance Index As A Marker Of Ecological Status | Moore et al. | *Journal Of Marine Research* | 2013 |
| Antimicrobial Test Of Five Ethnomedicinal Plants In An Ancestral Forest Area | Bandibas and Roxas | *Global Journal Of Environmental Science And Management-Gjesm* | 2017 |
| Approaches To Valuing Plant Medicines - The Economics Of Culture Or The Culture Of Economics | Brown | *Biodiversity And Conservation* | 1994 |
| Aquatic Ecosystems, Human Health, And Ecohydrology | Lara | *Treatise On Estuarine And Coastal Science, Vol 10: Ecohydrology And Restoration* | 2011 |

| | | | |
|---|---|---|---|
| Aquatic Eutrophication Promotes Pathogenic Infection In Amphibians | Johnson et al. | *Proceedings Of The National Academy Of Sciences Of The United States Of America* | 2007 |
| Aquatic Food Security: Insights Into Challenges And Solutions From An Analysis Of Interactions Between Fisheries, Aquaculture, Food Safety, Human Health, Fish And Human Welfare, Economy And Environment | Jennings et al. | *Fish And Fisheries* | 2016 |
| Aquatic Invasive Species And Emerging Infectious Disease Threats: A One Health Perspective | Conn | *Aquatic Invasions* | 2014 |
| Are Predators Good For Your Health? Evaluating Evidence For Top-Down Regulation Of Zoonotic Disease Reservoirs | Ostfeld and Holt | *Frontiers In Ecology And The Environment* | 2004 |
| Assessment Of Community Awareness And Risk Perceptions Of Zoonotic Causes Of Abortion In Cattle At Three Selected Livestock-Wildlife Interface Areas Of Zimbabwe | Ndengu et al. | *Epidemiology And Infection* | 2017 |
| Assessment Of Didecyldimethylammonium Chloride As A Ballast Water Treatment Method | van Slooten et al. | *Environmental Technology* | 2015 |
| Asymmetric Effects Of Native And Exotic Invasive Shrubs On Ecology Of The West Nile Virus Vector Culex Pipiens (Diptera: Culicidae) | Gardner et al. | *Parasites & Vectors* | 2015 |
| Avian Influenza H5N1 And The Wild Bird Trade In Hanoi, Vietnam | Brooks-Moizer et al. | *Ecology And Society* | 2009 |
| Bark In The Park: A Review Of Domestic Dogs In Parks | Weston et al. | *Environmental Management* | 2014 |
| Bat Rabies, Public Health And European Bat Conservation | Racey et al. | *Zoonoses And Public Health* | 2013 |
| Behavioural Differences: A Link Between Biodiversity And Pathogen Transmission | Dizney and Dearing | *Animal Behaviour* | 2016 |
| Biodiversity And Disease: A Synthesis Of Ecological Perspectives On Lyme Disease Transmission | Wood and Lafferty | *Trends In Ecology And Evolution* | 2013 |
| Biodiversity And Emerging Diseases | Maillard and Gonzalez | *Ann N Y Acad Sci* | 2006 |
| Biodiversity And Human Health: Evidence For Causality? | Hough | *Biodiversity And Conservation* | 2014 |
| Biodiversity And Leptospirosis Risk: A Case Of Pathogen Regulation? | Derne et al. | *Medical Hypotheses* | 2011 |
| Biodiversity Can Help Prevent Malaria Outbreaks In Tropical Forests | Laporta et al. | *Plos Neglected Tropical Diseases* | 2013 |
| Biodiversity Decreases Disease Through Predictable Changes In Host Community Competence | Johnson et al. | *Nature* | 2013 |
| Biodiversity Loss Affects Global Disease Ecology | Pongsiri et al. | *Bioscience* | 2009 |

| | | | |
|---|---|---|---|
| Biodiversity Loss And The Rise Of Zoonotic Pathogens | Ostfeld | *Clinical Microbiology And Infection* | 2009 |
| Biodiversity, Endemism, Sense Of Place, And Public Health: Inter-Relationships For Australian Inland Aquatic Systems | Horwitz et al. | *Ecosystem Health* | 2001 |
| Biological Control Agent For Mosquito Larvae: Review On The Killifish, Aphanius Dispar Dispar (Ruppel, 1829) | Al-Akel and Suliman | *African Journal Of Biotechnology* | 2011 |
| Biological Invaders Are Threats To Human Health: An Overview | Mazza et al. | *Ethology Ecology & Evolution* | 2014 |
| Biology And Impacts Of Pacific Island Invasive Species. 7. The Domestic Cat (Felis Catus) | Duffy and Capece | *Pacific Science* | 2012 |
| Broadening The Application Of Evolutionarily Based Genetic Pest Management | Gould | *Evolution* | 2008 |
| Can Integrating Wildlife And Livestock Enhance Ecosystem Services In Central Kenya? | Allan et al. | *Frontiers In Ecology And The Environment* | 2017 |
| Cascade Of Ecological Consequences For West Nile Virus Transmission When Aquatic Macrophytes Invade Stormwater Habitats | Mackay et al. | *Ecological Applications* | 2016 |
| Changing Patterns Of Emerging Zoonotic Diseases In Wildlife, Domestic Animals, And Humans Linked To Biodiversity Loss And Globalization | Aguirre | *Ilar J* | 2017 |
| Characteristics And Risk Perceptions Of Ghanaians Potentially Exposed To Bat-Borne Zoonoses Through Bushmeat | Kamins et al. | *Ecohealth* | 2015 |
| Chemical Contaminants In Feedlot Wastes: Concentrations, Effects And Attenuation | Khan et al. | *Environment International* | 2008 |
| Climate Change And Health: Transcending Silos To Find Solutions | Machalaba et al. | *Annals Of Global Health* | 2015 |
| Climate Change, Biodiversity, Ticks And Tick-Borne Diseases: The Butterfly Effect | Dantas-Torres | *International Journal For Parasitology-Parasites And Wildlife* | 2015 |
| Coastal Development And Precipitation Drive Pathogen Flow From Land To Sea: Evidence From A Toxoplasma Gondii And Felid Host System | VanWormer et al. | *Scientific Reports* | 2016 |
| Common Themes In Changing Vector-Borne Disease Scenarios. | Molyneux | *Trans R Soc Trop Med Hyg* | 2003 |
| Community Disassembly, Biodiversity Loss, And The Erosion Of An Ecosystem Service | Ostfeld and LoGiudice | *Ecology* | 2003 |
| Community-Based Conservation Reduces Sexual Risk Factors For Hiv Among Men. | Naidoo and Johnson | *Global Health* | 2013 |
| Comparison Between A Nature Reserve And Adjacent Communal Land In Xeric Succulent Thicket: An Indigenous Plant User'S Perspective | Fabricius and Burger | *South African Journal Of Science* | 1997 |
| Comparison Of Human And Southern Sea Otter (Enhydra Lutris Nereis) Health Risks | Adell et al. | *Water Research* | 2016 |

| | | | |
|---|---|---|---|
| For Infection With Protozoa In Nearshore Waters | | | |
| Consequences Of Non-Intervention For Infectious Disease In African Great Apes | Ryan and Walsh | *Plos One* | 2011 |
| Conservation Biology In Asia: The Major Policy Challenges | McNeely et al. | *Conservation Biology* | 2009 |
| Conservation Medicine And A New Agenda For Emerging Diseases | Daszak et al. | *Impact Of Ecological Changes On Tropical Animal Health And Disease Control* | 2004 |
| Conservation Medicine. | Deem et al. | *Ann N Y Acad Sci* | 2000 |
| Conservation Of Biodiversity As A Strategy For Improving Human Health And Well-Being | Kilpatrick et al. | *Philosophical Transactions Of The Royal Society B-Biological Sciences* | 2017 |
| Conservation, Development And The Management Of Infectious Disease: Avian Influenza In China, 2004-2012 | Wu and Perrings | *Philosophical Transactions Of The Royal Society B-Biological Sciences* | 2017 |
| Control And Prevention Of Rabies In Animals: Paradigm Shifts | Rupprecht et al. | *Developments In Biologicals* | 2006 |
| Cooperative Actions To Achieve Malaria Control Without The Use Of Ddt | Chanon et al. | *International Journal Of Hygiene And Environmental Health* | 2003 |
| Counting The Cost Of Vulture Decline - An Appraisal Of The Human Health And Other Benefits Of Vultures In India | Markandya et al. | *Ecological Economics* | 2008 |
| Current Status, Distribution, And Conservation Of Brown Bear (Ursidae) And Wild Canids (Gray Wolf, Golden Jackal, And Red Fox; Canidae) In Turkey | Ambarli et al. | *Turkish Journal Of Zoology* | 2016 |
| Declines In Large Wildlife Increase Landscape-Level Prevalence Of Rodent-Borne Disease In Africa | Young et al. | *Proceedings Of The National Academy Of Sciences Of The United States Of America* | 2014 |
| Deer Browse Resistant Exotic-Invasive Understory: An Indicator Of Elevated Human Risk Of Exposure To Ixodes Scapularis (Acari: Ixodidae) In Southern Coastal Maine Woodlands. | Elias et al. | *J Med Entomol* | 2006 |
| Deforestation - Effects On Vector-Borne Disease | Walsh et al. | *Parasitology* | 1993 |
| Demarcating Forest, Containing Disease: Land And Hiv/Aids In Southern Zambia | Frank and Unruh | *Population And Environment* | 2008 |
| Design And Management Of Free Water Surface Constructed Wetlands To Minimize Mosquito Production | Walton | *Wetlands Ecology And Management* | 2012 |

| | | | |
|---|---|---|---|
| Designing Graduate Training Programs In Conservation Medicine-Producing The Right Professionals With The Right Tools | Kaufman et al. | *Ecohealth* | 2008 |
| Detection Of Fecal Indicator Bacteria In Ship Ballast Water | Elcicek and Cakmakci | *Sigma Journal Of Engineering And Natural Sciences-Sigma Muhendislik Ve Fen Bilimleri Dergisi* | 2016 |
| Detection Of Hepatitis E Virus And Other Livestock-Related Pathogens In Iowa Streams | Givens et al. | *Science Of The Total Environment* | 2016 |
| Differences In Forest Use And Colonization By Neotropical Tree-Hole Damselffies (Odonata : Pseudostigmatidae): Implications For Forest Conversion | Fincke and Hedström | *Studies On Neotropical Fauna And Environment* | 2008 |
| Dioecious Caesalpinia Bonduc (L.) Roxb. Calls For Conservation In Burachapori Wildlife Sanctuary, Assam | Balasubramanian et al. | *Tropical Ecology* | 2016 |
| Disease Ecology, Health And The Environment: A Framework To Account For Ecological And Socio-Economic Drivers In The Control Of Neglected Tropical Diseases | Garchitorena et al. | *Philosophical Transactions Of The Royal Society B-Biological Sciences* | 2017 |
| Disease Emergence And Invasions | Hatcher et al. | *Functional Ecology* | 2012 |
| Disease Management Strategies For Wildlife | Wobeser et al. | *Rev Sci Tech* | 2002 |
| Disease Risk Analysis: A Tool For Primate Conservation Planning And Decision Making | Travis et al. | *American Journal Of Primatology* | 2006 |
| Does Biodiversity Protect Humans Against Infectious Disease? | Wood et al. | *Ecology* | 2014 |
| Does Deforestation Promote Or Inhibit Malaria Transmission In The Amazon? A Systematic Literature Review And Critical Appraisal Of Current Evidence | Lima et al. | *Philosophical Transactions Of The Royal Society B-Biological Sciences* | 2017 |
| Does Life History Mediate Changing Disease Risk When Communities Disassemble? | Joseph et al. | *Ecology Letters* | 2013 |
| Does The Impact Of Biodiversity Differ Between Emerging And Endemic Pathogens? The Need To Separate The Concepts Of Hazard And Risk | Hosseini et al. | *Philosophical Transactions Of The Royal Society B-Biological Sciences* | 2017 |
| Drivers For Occasional Spillover Event Of Ebola Virus | EFSA (European Food Safety Authority) | *Efsa Journal* | 2015 |
| Drivers Of Bushmeat Hunting And Perceptions Of Zoonoses In Nigerian Hunting Communities | Friant et al. | *Plos Neglected Tropical Diseases* | 2015 |
| Drug Development And Conservation Of Biodiversity In West And Central Africa: A | Schuster et al. | *Pharmaceutical Biology* | 1999 |

| | | | |
|---|---|---|---|
| Model For Collaboration With Indigenous People | | | |
| Dryland Salinity And The Ecology Of Ross River Virus: The Ecological Underpinnings Of The Potential For Transmission | Carver et al. | *Vector-Borne And Zoonotic Diseases* | 2009 |
| Ebola And The Decline Of Gorilla Gorilla Gorilla And Chimpanzee Pan Troglodytes Populations In Minkebe Forest, North-Eastern Gabon | Huijbregts et al. | *Oryx* | 2003 |
| Ebola In Great Apes - Current Knowledge, Possibilities For Vaccination, And Implications For Conservation And Human Health | Leendertz et al. | *Mammal Review* | 2017 |
| Echinococcus Multilocularis--A Zoonosis Of Anthropogenic Environments? | Romig et al. | *J Helminthol* | 2006 |
| Ecological And Toxicological Effects Of Inorganic Nitrogen Pollution In Aquatic Ecosystems: A Global Assessment | Camargo and Alonso | *Environment International* | 2006 |
| Ecological Correlates Of Risk And Incidence Of West Nile Virus In The United States | Allan et al. | *Oecologia* | 2009 |
| Ecological Determinants Of Health: Food And Environment On Human Health | Li | *Environmental Science And Pollution Research* | 2017 |
| Ecology And Biogeography Of Marine Parasites | Rohde | *Adv Mar Biol* | 2002 |
| Ecology And Demography Of Free-Roaming Domestic Dogs In Rural Villages Near Serengeti National Park In Tanzania | Czupryna | *Plos One* | 2016 |
| Ecosystem Services Connect Environmental Change To Human Health Outcomes | Bayles | *Ecohealth* | 2016 |
| Ecotourism With Utilization Of Wild Animals-Its Impact On Conservation Medicine And Risk Assessment In Hokkaido, Japan | Asakawa | *Ecotourism: Management, Development, And Impact* | 2010 |
| Effect Of Estuarine Wetland Degradation On Transport Of Toxoplasma Gondii Surrogates From Land To Sea | Shapiro | *Applied And Environmental Microbiology* | 2010 |
| Effect Of Landscape Structure On Anopheline Mosquito Density And Diversity In Northern Thailand: Implications For Malaria Transmission And Control | Overgaard et al. | *Landscape Ecology* | 2003 |
| Effects Of An Invasive Forest Pathogen On Abundance Of Ticks And Their Vertebrate Hosts In A California Lyme Disease Focus | Swei et al. | *Oecologia* | 2011 |
| Effects Of Environmental Change On Emerging Parasitic Diseases | Patz et al. | *International Journal For Parasitology* | 2000 |
| Effects Of Environmental Change On Malaria In The Amazon Region Of Brazil | Takken et al. | *Proceedings Of The Frontis Workshop On Environmental Change And Malaria Risk: Global And Local Implications* | 2005 |

| | | | |
|---|---|---|---|
| Effects Of Transparent Exopolymer Particles And Suspended Particles On The Survival Of Salmonella Enterica Serovar Typhimurium In Seawater | Davidson et al. | *Fems Microbiology Ecology* | 2015 |
| Efficiency Of A Subsurface Constructed Wetland System Using Native Southwestern Us Plants | Maschinski et al. | *Journal Of Environmental Quality* | 1999 |
| Emerging Diseases In Freshwater Systems | Okamura and Feist | *Freshwater Biology* | 2011 |
| Emerging Henipaviruses And Flying Foxes - Conservation And Management Perspectives | Breed et al. | *Biological Conservation* | 2006 |
| Emerging Infectious Disease Risk: Shared Drivers With Environmental Change | Machalaba and Karesh | *Revue Scientifique Et Technique-Office International Des Epizooties* | 2017 |
| Emerging Infectious Diseases : The Example Of The Indian Ocean Chikungunya Outbreak (2005-2006) | Flahault | *Bulletin De L Academie Nationale De Medecine* | 2007 |
| Emerging Infectious Diseases Of Wildlife And Species Conservation. | Medina-Vogel | *Microbiol Spectr* | 2013 |
| Emerging Issues And Challenges In Conservation Of Biodiversity In The Rangelands Of Tanzania | Kideghesho et al. | *Nature Conservation-Bulgaria* | 2013 |
| Emerging Risks From Bat Bushmeat In West Africa | Kamins et al. | *Trends In Game Meat Hygiene: From Forest To Fork* | 2014 |
| Emerging Urban Pests And Vector-Borne Diseases In Brazil | Campos | *Urban Pest Management: An Environmental Perspective* | 2011 |
| Empirical Evidence Of The Public Health Benefits Of Tropical Forest Conservation In Cambodia: A Generalised Linear Mixed-Effects Model Analysis | Pienkowski et al. | *The Lancet Planetary Health* | 2017 |
| Endoparasite Control Strategies: Implications For Biodiversity Of Native Fauna | Spratt | *International Journal For Parasitology* | 1997 |
| Engaging Research With Policy And Action: What Are The Challenges Of Responding To Zoonotic Disease In Africa? | Bardosh et al. | *Philosophical Transactions Of The Royal Society B-Biological Sciences* | 2017 |
| Environmental Change And Disease Dynamics: Effects Of Intensive Forest Management On Puumala Hantavirus Infection In Boreal Bank Vole Populations | Voutilainen et al. | *Plos One* | 2012 |
| Environmental Risk And Toxicology Of Human And Veterinary Waste Pharmaceutical Exposure To Wild Aquatic Host-Parasite Relationships | Morley et al. | *Environmental Toxicology And Pharmacology* | 2009 |
| Epidemiological Significance Of Bats (Chiroptera) In Europe, With Emphasis On Their Bloodsucking Ectoparasites As | Krisztina and Hornok | *Magyar Allatorvosok Lapja* | 2016 |

| | | | |
|---|---|---|---|
| Potential Transmitters Of Vector-Borne Pathogens | | | |
| Epidemiological Study Of Zoonoses Derived From Humans In Captive Chimpanzees | Kooriyama et al. | *Primates* | 2013 |
| Epidemiology Of Contagious Diseases, Ecology Of Mammals, Health, Management And Conservation Biology: Concluding Remarks. | Artois et al. | *Rev Sci Tech* | 1993 |
| Estimating Burdens Of Neglected Tropical Zoonotic Diseases On Islands With Introduced Mammals | de Wit et al. | *American Journal Of Tropical Medicine And Hygiene* | 2017 |
| Ethno-Botanical Study Of Medicinal Plants Of Paddar Valley Of Jammu And Kashmir, India | Gupta et al. | *African Journal Of Traditional Complementary And Alternative Medicines* | 2013 |
| Ethnobotany And Phytomedicine Of The Upper Nyong Valley Forest In Cameroon | Jiofack et al. | *African Journal Of Pharmacy And Pharmacology* | 2009 |
| Evidence For Human Streptococcus Pneumoniae In Wild And Captive Chimpanzees: A Potential Threat To Wild Populations | Köndgen et al. | *Scientific Reports* | 2017 |
| Evidence Of Land-Sea Transfer Of The Zoonotic Pathogen Campylobacter To A Wildlife Marine Sentinel Species | Baily et al. | *Molecular Ecology* | 2015 |
| Examining The Links Between Biodiversity And Human Health: An Interdisciplinary Research Initiative At The Us Environmental Protection Agency | Pongsiri and Roman | *Ecohealth* | 2007 |
| Exotic Grass Invasion Reduces Survival Of Amblyomma Americanum And Dermacentor Variabilis Ticks (Acari : Ixodidae) | Civitello et al. | *Journal Of Medical Entomology* | 2008 |
| Exploring Connections Among Nature, Biodiversity, Ecosystem Services, And Human Health And Well-Being: Opportunities To Enhance Health And Biodiversity Conservation | Sandifer et al. | *International Journal Of Sustainable Development And World Ecology* | 2015 |
| Exploring The Links Between Hiv/Aids And Forests In Malawi: Morbidity, Mortality, And Changing Dependence On Forest Resources | Timko | *Advances In Medical Sociology* | 2013 |
| Export Agriculture Is Feeding Malaria: A Cross-National Examination Of The Environmental And Social Causes Of Malaria Prevalence | Austin | *Population And Environment* | 2013 |
| Exposure To Infectious Agents In Dogs In Remote Coastal British Columbia: Possible Sentinels Of Diseases In Wildlife And Humans | Bryan | *Canadian Journal Of Veterinary Research-Revue Canadienne De Recherche Veterinaire* | 2011 |

| | | | |
|---|---|---|---|
| Extraction Of Non-Timber Forest Products As A Coping Strategy For Hiv/Aids-Afflicted Rural Households In South-Eastern Zimbabwe | Mutenje | *Ajar-African Journal Of Aids Research* | 2011 |
| Flying-Foxes In The Australian Urban Environment-Community Attitudes And Opinions. | Field et al. | *One Health* | 2015 |
| Food Safety Hazards And The Application Of The Principles Of The Hazard Analysis And Critical Control Point (Haccp) System For Their Control In Aquaculture Production | Reilly et al. | *Aquaculture Research* | 1997 |
| Foraging Behaviour And Landscape Utilisation By The Endangered Golden-Crowned Flying Fox (Acerodon Jubatus), The Philippines | de Jong et al. | *Plos One* | 2013 |
| Forest Cover Associated With Improved Child Health And Nutrition: Evidence From The Malawi Demographic And Health Survey And Satellite Data | Johnson et al. | *Global Health: Science And Practice* | 2013 |
| Forests, Women And Health: Opportunities And Challenges For Conservation | Wan et al. | *International Forestry Review* | 2011 |
| Frequency Of Zoonotic Bacteria Among Illegally Traded Wild Birds In Rio De Janeiro | Matias et al. | *Brazilian Journal Of Microbiology* | 2016 |
| Freshwater Ecology And Biodiversity In The Tropics: What Did We Learn From 30 Years Of Onchocerciasis Control And The Associated Biomonitoring Of West African Rivers? | Lévêque et al. | *Hydrobiologia* | 2003 |
| Functional Consequences Of Invasive Species In Coastal And Estuarine Systems | Levin and Crooks | *Treatise On Estuarine And Coastal Science, Vol 7: Functioning Of Ecosystems At The Land-Ocean Interface* | 2011 |
| Fundamental Knowledge About Insect Reproduction: Essential To Develop Sustainable Pest Management | van Lenteren | *Invertebrate Reproduction & Development* | 1999 |
| Future Novel Threats And Opportunities Facing Uk Biodiversity Identified By Horizon Scanning | Sutherland et al. | *Journal Of Applied Ecology* | 2008 |
| Future Of Keeping Pet Reptiles And Amphibians: Towards Integrating Animal Welfare, Human Health And Environmental Sustainability | Pasmans et al. | *Veterinary Record* | 2017 |
| Gastrointestinal Bacterial Transmission Among Humans, Mountain Gorillas, And Livestock In Bwindi Impenetrable National Park, Uganda | Rwego et al. | *Conservation Biology* | 2008 |
| Gastrointestinal Parasite Infections And Self-Medication In Wild Chimpanzees Surviving In Degraded Forest Fragments Within An Agricultural Landscape Mosaic In Uganda | McLennan et al. | *Plos One* | 2017 |

| | | | |
|---|---|---|---|
| Genetic Evolution Of Influenza H9N2 Viruses Isolated From Various Hosts In China From 1994 To 2013 | Li et al. | *Emerging Microbes & Infections* | 2017 |
| Global Change And Human Susceptibility To Disease | Daily and Ehrlich | *Annual Review Of Energy And The Environment* | 1996 |
| Global Change And Human Vulnerability To Vector-Borne Diseases | Sutherst | *Clinical Microbiology Reviews* | 2004 |
| Global Diversity Of Copepods (Crustacea : Copepoda) In Freshwater | Boxshall and Defaye | *Hydrobiologia* | 2008 |
| Good Governance In `One Health' Approaches | Lanford and Nunn | *Revue Scientifique Et Technique-Office International Des Epizooties* | 2012 |
| Growing Population And Ecosystem Change Increase Human Schistosomiasis Around Lake Malawi | Van Bocxlaer | *Trends In Parasitology* | 2014 |
| Guidelines To Strengthen The Program Of Malaria In Remote Areas With Indigenous Population In The Caura, Venezuela | Bevilacqua et al. | *Boletin De Malariologia Y Salud Ambiental* | 2009 |
| Habitat Properties Are Key Drivers Of Borrelia Burgdorferi (S.L.) Prevalence In Ixodes Ricinus Populations Of Deciduous Forest Fragments | Ehrmann et al. | *Parasites & Vectors* | 2018 |
| Habituating The Great Apes: The Disease Risks | Woodford et al. | *Oryx* | 2002 |
| Halophytic Life In Brazilian Salt Flats: Biodiversity, Uses And Threats | Costa and Herrera | *Sabkha Ecosystems Vol V: The Americas* | 2016 |
| Hendra In The News: Public Policy Meets Public Morality In Times Of Zoonotic Uncertainty | Degeling and Kerridge | *Social Science & Medicine* | 2013 |
| Henipaviruses: Emerging Paramyxoviruses Associated With Fruit Bats | Field et al. | *Wildlife And Emerging Zoonotic Diseases: The Biology, Circumstances, And Consequences Of Cross-Species Transmission* | 2007 |
| High-Resolution Gps Tracking Of Lyle'S Flying Fox Between Temples And Orchards In Central Thailand | Weber et al. | *Journal Of Wildlife Management* | 2015 |
| Hiv And Aids Among Fisherfolk: A Threat To Responsible Fisheries? | Allison and Seeley | *Fish And Fisheries* | 2004 |
| Hiv/Aids: A Risk To The Social And Economic Sustainability Of Forestry In Sub-Saharan Africa? | Topouzis | *International Forestry Review* | 2007 |
| How Do Hiv And Aids Impact The Use Of Natural Resources By Poor Rural Populations? The Case Of Wild Animal Products | Kaschulal and Shackleton | *South African Journal Of Science* | 2012 |
| How The Biodiversity Sciences May Aid Biological Tools And Ecological Engineering To Assess The Impact Of Climatic Changes | Morand and Guégan | *Revue Scientifique Et Technique-Office International Des Epizooties* | 2008 |

| | | | |
|---|---|---|---|
| Human And Environmental Health Risks And Benefits Associated With Use Of Urban Stormwater | Jiang et al. | *Wiley Interdisciplinary Reviews-Water* | 2015 |
| Human Disease Hinders Anti-Poaching Efforts In Indian Nature Reserves | Velho et al. | *Biological Conservation* | 2011 |
| Human Health Alters The Sustainability Of Fishing Practices In East Africa | Fiorella et al. | *Proceedings Of The National Academy Of Sciences Of The United States Of America* | 2017 |
| Human Health Impacts Of Ecosystem Alteration | Myers et al. | *Proceedings Of The National Academy Of Sciences Of The United States Of America* | 2013 |
| Human Health-Related Ecosystem Services Of Avian-Dense Coastal Wetlands Adjacent To A Western Lake Erie Swimming Beach | Rea et al. | *Ecohealth* | 2015 |
| Human Infectious Disease Burdens Decrease With Urbanization But Not With Biodiversity | Wood et al. | *Philosophical Transactions Of The Royal Society B-Biological Sciences* | 2017 |
| Human Schistosomiasis In Wetlands In Southern Africa | Appleton and Madsen | *Wetlands Ecology And Management* | 2012 |
| Human-Resources Strategies For Managing Hiv/Aids: The Case Of The South African Forestry Industry | Gow and Grant | *Ajar-African Journal Of Aids Research* | 2010 |
| Human-Wildlife Interactions And Zoonotic Transmission Of Echinococcus Multilocularis | Hegglin et al. | *Trends In Parasitology* | 2015 |
| Hunting Of Flying Foxes And Perception Of Disease Risk In Indonesian Borneo | Harrison et al. | *Biological Conservation* | 2011 |
| Hydrologic And Vegetative Removal Of Cryptosporidium Parvum, Giardia Lamblia, And Toxoplasma Gondii Surrogate Microspheres In Coastal Wetlands | Hogan et al. | *Applied And Environmental Microbiology* | 2013 |
| Hydrological Modelling Of Toxoplasma Gondii Oocysts Transport To Investigate Contaminated Snowmelt Runoff As A Potential Source Of Infection For Marine Mammals In The Canadian Arctic | Simon et al. | *Journal Of Environmental Management* | 2013 |
| Identification Of Mycobacterium Species From Apparently Healthy Freshwater Aquarium Fish Using Partial Sequencing And Pcr- Rflp Analysis Of Heat Shock Protein ( Hsp65) Gene | Shukla et al. | *Journal Of Applied Ichthyology* | 2014 |
| Impact Of Ballast Water On Environmental Health | Husna et al. | *Advanced Science Letters* | 2017 |
| Impacts Of An Introduced Forest Pathogen On The Risk Of Lyme Disease In California | Swei et al. | *Vector-Borne And Zoonotic Diseases* | 2012 |
| Impacts Of Biodiversity On The Emergence And Transmission Of Infectious Diseases | Keesing et al. | *Nature* | 2010 |

| | | | |
|---|---|---|---|
| Impacts Of The Creation, Expansion And Management Of English Wetlands On Mosquito Presence And Abundance - Developing Strategies For Future Disease Mitigation | Medlock and Vaux | *Parasites & Vectors* | 2015 |
| In Vitro Investigation Of Brazilian Cerrado Plant Extract Activity Against Plasmodium Falciparum, Trypanosoma Cruzi And T-Brucei Gambiense | Charneau et al. | *Natural Product Research* | 2016 |
| Increased Avian Diversity Is Associated With Lower Incidence Of Human West Nile Infection: Observation Of The Dilution Effect | Swaddle and Calos | *Plos One* | 2008 |
| Individual And Environmental Factors Associated With The Seroprevalence Of Borrelia Burgdorferi In Belgian Farmers And Veterinarians. | De Keukeleire et al. | *Infect Ecol Epidemiol* | 2016 |
| Infections And Diseases In Wildlife By Non-Native Organisms | Morand | *Impact Of Biological Invasions On Ecosystem Services* | 2017 |
| Influence Of Deforestation, Logging, And Fire On Malaria In The Brazilian Amazon | Hahn et al. | *Plos One* | 2014 |
| Influence Of Protected Areas On Malaria Prevalence In Sub-Saharan Africa | Taber and Smithwick | *Applied Geography* | 2015 |
| Injudicious And Excessive Use Of Antibiotics: Public Health And Salmon Aquaculture In Chile | Millanao et al. | *Revista Medica De Chile* | 2011 |
| Integrative Approaches To The Study Of Primate Infectious Disease: Implications For Biodiversity Conservation And Global Health | Gillespie et al. | *American Journal Of Physical Anthropology* | 2008 |
| Integrative Molecular Phylogeography In The Context Of Infectious Diseases On The Human-Animal Interface | Gray and Salemi | *Parasitology* | 2012 |
| Intensifying Poultry Production Systems And The Emergence Of Avian Influenza In China: A `One Health/Ecohealth' Epitome | Gilbert et al. | *Archives Of Public Health* | 2017 |
| Interacting Effects Of Land Use And Climate On Rodent-Borne Pathogens In Central Kenya | Young et al. | *Philosophical Transactions Of The Royal Society B-Biological Sciences* | 2017 |
| International Health And Emerging Infectious Diseases. | Ebomoyi and Ebomoyi | *J Health Hum Serv Adm* | 2000 |
| Invasions Of Vector-Borne Diseases Driven By Transportation And Climate Change | van der Weijden et al. | *Emerging Pests And Vector-Borne Diseases In Europe* | 2007 |
| Invasive Honeysuckle Eradication Reduces Tick-Borne Disease Risk By Altering Host Dynamics | Allan et al. | *Proceedings Of The National Academy Of Sciences Of The United States Of America* | 2010 |
| Inventorization Of Some Ayurvedic Plants And Their Ethnomedicinal Use In Kakrajhore Forest Area Of West Bengal | Biswas et al. | *Journal Of Ethnopharmacology* | 2017 |
| Joining The Dots - Understanding The Complex Interplay Between The Values We Place On Wildlife, Biodiversity | Ryser-Degiorgis et al. | *Schweizer Archiv Fur Tierheilkunde* | 2015 |

| | | | |
|---|---|---|---|
| Conservation, Human And Animal Health: A Review | | | |
| Land Cover Variation And West Nile Virus Prevalence: Patterns, Processes, And Implications For Disease Control | Ezenwa et al. | *Vector-Borne And Zoonotic Diseases* | 2007 |
| Landscape Dynamics In The Wildland-Urban Interface | Zipperer | *Urban-Rural Interfaces: Linking People And Nature* | 2012 |
| Landscape Variation Influences Trophic Cascades In Dengue Vector Food Webs. | Weterings et al. | *Sci Adv* | 2018 |
| Large-Scale Removal Of Invasive Honeysuckle Decreases Mosquito And Avian Host Abundance | Gardner et al. | *Ecohealth* | 2017 |
| Larvivorous Fish For Preventing Malaria Transmission | Walshe et al. | *Cochrane Database Of Systematic Reviews* | 2013 |
| Legal Issues In Sharing The Benefits Of Biodiversity Prospecting. | Mays and Mazan | *J Ethnopharmacol* | 1996 |
| Leopards Provide Public Health Benefits In Mumbai, India | Braczkowski et al. | *Frontiers In Ecology And The Environment* | 2018 |
| Leptospirosis Risk Increases With Changes In Species Composition Of Rat Populations | Theuerkauf et al. | *Naturwissenschaften* | 2013 |
| Linking Environmental Nutrient Enrichment And Disease Emergence In Humans And Wildlife | Johnson et al. | *Ecological Applications* | 2010 |
| Linking Future Ecosystem Services And Future Human Well-Being | Butler and Oluoch-Kosura | *Ecology And Society* | 2006 |
| Lyme Disease Ecology In A Changing World: Consensus, Uncertainty And Critical Gaps For Improving Control | Kilpatrick et al. | *Philosophical Transactions Of The Royal Society B-Biological Sciences* | 2017 |
| Lyme-Disease And Conservation | Ginsberg | *Conservation Biology* | 1994 |
| Main Aspects Of The Pollution In Brazilian Rivers By Pesticides | Dellamatrice and Monteiro | *Revista Brasileira De Engenharia Agricola E Ambiental* | 2014 |
| Malaria Control In Amerindian Communities Of Venezuela Strengthening Ecohealth Practice Throughout Conservation Science And Capability Approach | Bevilacqua et al. | *Ecohealth* | 2015 |
| Malaria On The Amazonian Frontier: Transmission Dynamics, Risk Factors, Spatial Distribution, And Prospects For Control | da Silva-Nunes et al. | *American Journal Of Tropical Medicine And Hygiene* | 2008 |
| Mammal Decline, Linked To Invasive Burmese Python, Shifts Host Use Of Vector Mosquito Towards Reservoir Hosts Of A Zoonotic Disease | Hoyer et al. | *Biology Letters* | 2017 |
| Management Of Wildlife-Human Conflicts In Israel: A Wide Variety Of Vertebrate Pest Problems In A Difficult And Compact Environment | Nemtzov | *Proceedings Of The 20Th Vertebrate Pest Conference* | 2002 |
| Managing Emerging Diseases Borne By Fruit Bats (Flying Foxes), With Particular | Mackenzie et al. | *Journal Of Applied Microbiology* | 2003 |

| | | | |
|---|---|---|---|
| Reference To Henipaviruses And Australian Bat Lyssavirus | | | |
| Marine Mammals As Sentinel Species For Oceans And Human Health | Bossart | *Veterinary Pathology* | 2011 |
| Medicinal Plants At Rio Jauaperi, Brazilian Amazon: Ethnobotanical Survey And Environmental Conservation | Pedrollo et al. | *Journal Of Ethnopharmacology* | 2016 |
| Medicinal Plants In Forest Stands Around The Bay Of Rigny-Antsiranana In Madagascar | Rakotonandrasana et al. | *Bois Et Forets Des Tropiques* | 2017 |
| Medicinal Plants Used By Women From Agnalazaha Littoral Forest (Southeastern Madagascar) | Razafindraibe et al. | *Journal Of Ethnobiology And Ethnomedicine* | 2013 |
| Medicinal Plants Used For Management Of Malaria Among The Luhya Community Of Kakamega East Sub-County, Kenya | Mukungu et al. | *Journal Of Ethnopharmacology* | 2016 |
| Medicinal Plants Used In Traditional Medicine By Oromo People, Ghimbi District, Southwest Ethiopia | Abera | *Journal Of Ethnobiology And Ethnomedicine* | 2014 |
| Medicinal Utilization Of Exotic Plants By Bapedi Traditional Healers To Treat Human Ailments In Limpopo Province, South Africa | Semenya et al. | *Journal Of Ethnopharmacology* | 2012 |
| Microbiomes, Metagenomics, And Primate Conservation: New Strategies, Tools, And Applications | Stumpf et al. | *Biological Conservation* | 2016 |
| Mitigating An Elephantine Epidemic: Gendered Space For Hiv/Aids Outreach Through Namibian Conservancies | DeMotts | *Population And Environment* | 2008 |
| Modeling The Impact Of Ebola And Bushmeat Hunting On Western Lowland Gorillas | Rizkalla et al. | *Ecohealth* | 2007 |
| Modeling The Impacts Of Global Warming On Predation And Biotic Resistance: Mosquitoes, Damselflies And Avian Malaria In Hawaii | Hobbelen et al. | *Theoretical Ecology* | 2013 |
| Molecular Epidemiology Of Cross-Species Giardia Duodenalis Transmission In Western Uganda | Johnson et al. | *Plos Neglected Tropical Diseases* | 2010 |
| Mosquito Vector Diversity Across Habitats In Central Thailand Endemic For Dengue And Other Arthropod-Borne Diseases | Thongsripong et al. | *Plos Neglected Tropical Diseases* | 2013 |
| Multiple Use Patterns Of Medicinal Trees In An Urban Forest In Nairobi, Kenya | Furukawa et al. | *Urban Forestry & Urban Greening* | 2016 |
| Multiple-Host Pathogens In Domestic Hunting Dogs In Nicaragua's Bosawas Biosphere Reserve | Fiorello et al. | *Acta Tropica* | 2017 |
| Multistate Evaluation Of Microbial Water And Sediment Quality From Agricultural Recovery Basins | Partyka et al. | *Journal Of Environmental Quality* | 2016 |
| Mycobacteriosis In Fishes: A Review | Gauthier and Rhodes | *Veterinary Journal* | 2009 |

| | | | |
|---|---|---|---|
| Nature'S Care: Diarrhea, Watershed Protection, And Biodiversity Conservation In Flores, Indonesia | Pattanayak and Wendland | *Biodiversity And Conservation* | 2007 |
| Navigating Parasite Webs And Parasite Flow: Emerging And Re-Emerging Parasitic Zoonoses Of Wildlife Origin | Polley | *International Journal For Parasitology* | 2005 |
| Nearly 400 Million People Are At Higher Risk Of Schistosomiasis Because Dams Block The Migration Of Snail-Eating River Prawns | Sokolow et al. | *Philosophical Transactions Of The Royal Society B-Biological Sciences* | 2017 |
| Neotropical Zoonotic Parasites In Bush Dogs (Speothos Venaticus) From Upper Parana Atlantic Forests In Misiones, Argentina | Vizcaychipi et al. | *Vector-Borne And Zoonotic Diseases* | 2016 |
| Nonnative Fish To Control Aedes Mosquitoes: A Controversial, Harmful Tool | Azevedo-Santos et al. | *Bioscience* | 2017 |
| Novel Viral Encephalitides Associated With Bats (Chiroptera) - Host Management Strategies | Field et al. | *Archives Of Virology* | 2004 |
| Null Expectations For Disease Dynamics In Shrinking Habitat: Dilution Or Amplification? | Faust et al. | *Philosophical Transactions Of The Royal Society B-Biological Sciences* | 2017 |
| Observations Of Sylvatic Rabies In Northern Argentina During Outbreaks Of Paralytic Cattle Rabies Transmitted By Vampire Bats (Desmodus Rotundus) | Delpietro et al. | *Journal Of Wildlife Diseases* | 2009 |
| Oceans And Human Health: Emerging Public Health Risks In The Marine Environment | Fleming et al. | *Marine Pollution Bulletin* | 2006 |
| Of Mice And Men Lyme Disease And Biodiversity | Granter et al. | *Perspectives In Biology And Medicine* | 2014 |
| Oil doom and AIDS boom in the Niger Delta Region of Nigeria | Udonwa et al. | *Rural Remote Health* | 2004 |
| One Health Messaging About Bats And Rabies: How Framing Of Risks, Benefits And Attributions Can Support Public Health And Wildlife Conservation Goals | Lu et al. | *Wildlife Research* | 2017 |
| One Health, Emerging Infectious Diseases And Wildlife: Two Decades Of Progress? | Cunningham et al. | *Philosophical Transactions Of The Royal Society B-Biological Sciences* | 2017 |
| Opportunities And Challenges With Growing Wildlife Populations And Zoonotic Diseases In Sweden | Thulin et al. | *European Journal Of Wildlife Research* | 2015 |
| Optimization Of Human, Animal, And Environmental Health By Using The One Health Approach | Sleeman et al. | *Journal Of Veterinary Science* | 2017 |
| Oral Immunization Against Rabies: Afterthoughts And Foresight. | Wandeler | *Schweizer Archiv Fur Tierheilkunde* | 2000 |
| Parasites And The Conservation Of Small Populations: The Case Of Baylisascaris Procyonis. | Page | *Int J Parasitol Parasites Wildl* | 2013 |

| | | | |
|---|---|---|---|
| Pathogen Transmission From Humans To Great Apes Is A Growing Threat To Primate Conservation. | Dunay et al. | *Ecohealth* | 2018 |
| Patterns Of Extinction In Prairie Dog Metapopulations: Plague Outbreaks Follow El Nino Events | Stapp et al. | *Frontiers In Ecology And The Environment* | 2004 |
| Perceived Vaccination Status In Ecotourists And Risks Of Anthropozoonoses | Muehlenbein et al. | *Ecohealth* | 2008 |
| Perspectives On Advancing Preventative Medicine Through Vaccinology At The Comparative Veterinary, Human And Conservation Medicine Interface: Not Missing The Opportunities | Nara et al. | *Vaccine* | 2008 |
| Perspectives On Wastewater Treatment Wetlands And Waterbird Conservation | Murray and Hamilton | *Journal Of Applied Ecology* | 2010 |
| Peruvian Medicinal Plant Sources Of New Pharmaceuticals (International Cooperative Biodiversity Group-Peru) | Lewis et al. | *Pharmaceutical Biology* | 1999 |
| Plant Resources And Social Preferences In Rural Environment Within Senegal | Diop et al. | *Bois Et Forets Des Tropiques* | 2011 |
| Population, Health, And Environment Situational Analysis For The Saadani National Park Area, Tanzania | Torell et al. | *Ocean & Coastal Management* | 2012 |
| Predicting Potential Ranges Of Primary Malaria Vectors And Malaria In Northern South America Based On Projected Changes In Climate, Land Cover And Human Population | Alimi et al. | *Parasites & Vectors* | 2015 |
| Prevalence And Climatic Associated Factors Of Cryptosporidium Sp Infections In Savanna Chimpanzees From Ugalla, Western Tanzania | Gonzalez-Moreno et al. | *Parasitology Research* | 2013 |
| Prevalence And Risk Factors For Infection Of Bovine Tuberculosis In Indigenous Cattle In The Serengeti Ecosystem, Tanzania. | Katale et al. | *Bmc Veterinary Research* | 2013 |
| Prevalence And Risk Factors For Viral Exposure In Rural Dogs Around Protected Areas Of The Atlantic Forest | Curi et al. | *Bmc Veterinary Research* | 2016 |
| Prevalence Of Cryptosporidium And Other Enteric Parasites Among Wild Non-Human Primates In Polonnaruwa, Sri Lanka | Ekanayake et al. | *Am J Trop Med Hyg* | 2006 |
| Prevention Educational Program Of Human Rabies Transmitted By Bats In Rain Forest Preserved Area Of Southern Brazilian Coast | Kikuti et al. | *Zoonoses And Public Health* | 2011 |
| Primates And The Ecology Of Their Infectious Diseases: How Will Anthropogenic Change Affect Host-Parasite Interactions? | Chapman et al. | *Evolutionary Anthropology* | 2005 |
| Pteridophytes: Evolutionary Boon As Medicinal Plants | Goswami et al. | *Plant Genetic Resources - Characterization And Utilization* | 2016 |
| Public Health And Epidemiological Considerations For Avian Influenza Risk Mapping And Risk Assessment | Dudley | *Ecology And Society* | 2008 |

| | | | |
|---|---|---|---|
| Public Health Impacts Of Ecosystem Change In The Brazilian Amazon | Bauch et al. | *Proceedings Of The National Academy Of Sciences Of The United States Ofamerica* | 2015 |
| Public Health Perspectives On Aquaculture. | Gormaz et al. | *Current Environ Health Rep* | 2014 |
| Pythons, Parasites, And Pests: Anthropogenic Impacts On Sarcocystis (Sarcocystidae) Transmission In A Multi-Host System | Devan-Song et al. | *Biotropica* | 2017 |
| Quassia ``Biopiracy'', Case And The Nagoya Protocol: A Researcher'S Perspective | Bourdy et al. | *Journal Of Ethnopharmacology* | 2017 |
| Rabies Elimination Research: Juxtaposing Optimism, Pragmatism And Realism | Cleaveland and Hampson | *Proceedings Of The Royal Society B-Biological Sciences* | 2017 |
| Raccoons In Europe: Disease Hazards Due To The Establishment Of An Invasive Species | Beltrán-Beck et al. | *European Journal Of Wildlife Research* | 2012 |
| Reconstructing The Emergence Of A Lethal Infectious Disease Of Wildlife Supports A Key Role For Spread Through Translocations By Humans | Price et al. | *Proceedings Of The Royal Society B-Biological Sciences* | 2016 |
| Red Fox And Tick-Borne Encephalitis (Tbe) In Humans: Can Predators Influence Public Health? | Haemig et al. | *Scandinavian Journal Of Infectious Diseases* | 2008 |
| Reduced Transmission Of Human Schistosomiasis After Restoration Of A Native River Prawn That Preys On The Snail Intermediate Host | Sokolow et al. | *Proceedings Of The National Academy Of Sciences Of The United States Of America* | 2015 |
| Relationship Between Burden Of Infection In Ungulate Populations And Wildlife/Livestock Interfaces | Caron et al. | *Epidemiology And Infection* | 2013 |
| Removal Of Enteric Viruses And Escherichia Coli From Municipal Treated Effluent By Zebra Mussels | Mezzanotte et al. | *Science Of The Total Environment* | 2016 |
| Reproduction And Nutritional Stress Are Risk Factors For Hendra Virus Infection In Little Red Flying Foxes (Pteropus Scapulatus) | Plowright et al. | *Proceedings Of The Royal Society B-Biological Sciences* | 2008 |
| Resistance Is Not Futile: It Shapes Insecticide Discovery. | Hardy | *Insects* | 2014 |
| Responses To Malaria Incidence In The Sango Bay Forest Reserve, Uganda | Galabuzi et al. | *Human Ecology* | 2016 |
| Role Of India'S Wildlife In The Emergence And Re-Emergence Of Zoonotic Pathogens, Risk Factors And Public Health Implications. | Singh and Gajadhar | *Acta Tropica* | 2014 |
| Roosting Behaviour And Habitat Selection Of Pteropus Giganteus Reveal Potential Links To Nipah Virus Epidemiology | Hahn et al. | *Journal Of Applied Ecology* | 2014 |
| Royal Society Of Tropical Medicine And Hygiene Meeting At Manson House, London, 20 March 1997 - Epidemiology And | Cleaveland | *Transactions Of The Royal Society Of Tropical Medicine And Hygiene* | 1998 |

| | | | |
|---|---|---|---|
| Control Of Rabies - The Growing Problem Of Rabies In Africa | | | |
| Salmon Aquaculture And Antimicrobial Resistance In The Marine Environment | Buschmann et al. | *Plos One* | 2012 |
| Salt Marsh As Culex Salinarius Larval Habitat In Coastal New York | Rochlin et al. | *Journal Of The American Mosquito Control Association* | 2008 |
| Saving The Rainforest Through Health Care: Medicine As Conservation In Borneo | Ali and Jacobs | *International Journal Of Occupational And Environmental Health* | 2007 |
| Scale-Dependent Effects Of Nonnative Plant Invasion On Host-Seeking Tick Abundance | Adalsteinsson et al. | *Ecosphere* | 2016 |
| Schistosomiasis In Lake Malawi: Relationship Of Fish And Intermediate Host Density To Prevalence Of Human Infection | Stauffer et al. | *Ecohealth* | 2006 |
| Science And The Conservation Of Bats: Where To Next? | Fenton | *Wildlife Society Bulletin* | 2003 |
| Science-Policy Challenges For Biodiversity, Public Health And Urbanization: Examples From Belgium | Keune et al. | *Environmental Research Letters* | 2013 |
| Seafood Insecurity, Bush Meat Consumption, And Public Health Emergency In West Africa: Did We Miss The Early Warning Signs Of An Ebola Epidemic? | Khan and Sesay | *Maritime Studies* | 2015 |
| Seagrass Ecosystems Reduce Exposure To Bacterial Pathogens Of Humans, Fishes, And Invertebrates | Lamb et al. | *Science* | 2017 |
| Seasonal Dynamics Of Microbial Community In An Aquaculture System For Nile Tilapia (Oreochromis Niloticus) | Resende et al. | *Aquaculture Research* | 2015 |
| Seasonal Genetic Partitioning In The Neotropical Malaria Vector, Anopheles Darlingi | Angêlla et al. | *Malaria Journal* | 2014 |
| Significance Of Endangered And Threatened Plant Natural Products In The Control Of Human Disease | Ibrahim et al. | *Proceedings Of The National Academy Of Sciences Of The United States Of America* | 2013 |
| Sleeping Sickness And Its Relationship With Development And Biodiversity Conservation In The Luangwa Valley, Zambia | Anderson et al. | *Parasites & Vectors* | 2015 |
| Socioeconomic And Health Implications Of Human-Wildlife Interactions In Nthongoni, Eastern Kenya | Mwangi et al. | *African Journal Of Wildlife Research* | 2016 |
| Spatial Characterization Of Colonies Of The Flying Fox Bat, A Carrier Of Nipah Virus In Thailand | Thanapongtharm et al. | *Bmc Veterinary Research* | 2015 |
| Spatial Overlap Between People And Non-Human Primates In A Fragmented Landscape | Paige et al. | *Ecohealth* | 2017 |

| | | | |
|---|---|---|---|
| Spatiotemporal Analysis Of Cryptosporidium Species/Genotypes And Relationships With Other Zoonotic Pathogens In Surface Water From Mixed-Use Watersheds | Wilkes et al. | *Applied And Environmental Microbiology* | 2013 |
| Special Challenges Of Maintaining Wildlife In Captivity In Europe And Asia | Kirkwood | *Revue Scientifique Et Technique De L Office International Des Epizooties* | 1996 |
| Strategies For Effective Mosquito Control In Constructed Treatment Wetlands | Knight et al. | *Ecological Engineering* | 2003 |
| Surrogate Hosts: Hunting Dogs And Recolonizing Grey Wolves Share Their Endoparasites | Lesniak et al. | *International Journal For Parasitology-Parasites And Wildlife* | 2017 |
| Surrounding Land Use Significantly Influences Adult Mosquito Abundance And Species Richness In Urban Mangroves | Claflin and Webb | *Wetlands Ecology And Management* | 2017 |
| Sustainability Of Current Agriculture Practices, Community Perception, And Implications For Ecosystem Health: An Indian Study | Sarkar et al. | *Ecohealth* | 2011 |
| Sustainable Food Systems For Optimal Planetary Health | Canavan et al. | *Transactions Of The Royal Society Of Tropical Medicine And Hygiene* | 2017 |
| Temporal Association Between Land-Based Runoff Events And California Sea Otter (Enhydra Lutris Nereis) Protozoal Mortalities | Shapiro et al. | *Journal Of Wildlife Diseases* | 2012 |
| Temporal Variability In Domestic Point Source Discharges And Their Associated Impact On Receiving Waters | Richards et al. | *Science Of The Total Environment* | 2016 |
| The Colorado Front Range Prairie Dog Technical Workshop: An Overview And Summary | Witmer and Hoffmann | *Twentieth Vertebrate Pest Conference, Proceedings* | 2002 |
| The Concentration Of Swine Production. Effects On Swine Health, Productivity, Human Health, And The Environment. | Donham | *Vet Clin North Am Food Anim Pract* | 2000 |
| The Contribution Of Predators And Scavengers To Human Well-Being | O'Bryan et al. | *Nature Ecology And Evolution* | 2018 |
| The Ecology And Emergence Of Diseases In Fresh Waters | Johnson and Paull | *Freshwater Biology* | 2011 |
| The Ecology Of Infectious Disease: Effects Of Host Diversity And Community Composition On Lyme Disease Risk | LoGiudice et al. | *Proceedings Of The National Academy Of Sciences Of The United States Of America* | 2003 |
| The Environment As A Strategic Healthcare Partner | Gaffikin | *Current Opinion In Obstetrics & Gynecology* | 2013 |
| The Environmental And Social Influences Of Hiv/Aids In Sub-Saharan Africa: A Focus On Rural Communities | Oramasionwu et al. | *Int J Environ Res Public Health* | 2011 |
| The Human Health And Conservation Relevance Of Food Taboos In Northeastern Madagascar | Golden and Comaroff | *Ecology And Society* | 2015 |
| The Integration Of Biodiversity Into One Health | Romanelli et al. | *Revue Scientifique Et Technique-Office* | 2014 |

| | | | |
|---|---|---|---|
| | | *International Des Epizooties* | |
| The Interactions Between Humans And Mammals In Africa In Relation To Conservation - A Review | Happold | *Biodiversity And Conservation* | 1995 |
| The Interconnection Between The Built Environment Ecology And Health | Koren and Butler | *Environmental Security And Environmental Management: The Role Of Risk Assessment* | 2006 |
| The Medical Importance Of Riceland Mosquitoes And Their Control Using Alternatives To Chemical Insecticides. | Lacey and Lacey | *J Am Mosq Control Assoc Suppl* | 1990 |
| The Miombo Woodlands At The Cross Roads: Potential Threats, Sustainable Livelihoods, Policy Gaps And Challenges | Syampungani et al. | *Natural Resources Forum* | 2009 |
| The Nutritional Role Of Indigenous Foods In Mitigating The Hiv/Aids Crisis In West And Central Africa | Kengni et al. | *International Forestry Review* | 2004 |
| The Prevalence Of Antibiotic-Resistant Bacteria (Arb) In Waters Of The Lower Ballona Creek Watershed, Los Angeles County, California | Kawecki et al. | *Environmental Monitoring And Assessment* | 2017 |
| The Relationship Between Physiological Stress And Wildlife Disease: Consequences For Health And Conservation | Hing et al. | *Wildlife Research* | 2016 |
| The Rise Of Non-Native Vectors And Reservoirs Of Human Diseases | Rabitsch et al. | *Impact Of Biological Invasions On Ecosystem Services* | 2017 |
| The River Ruhr - An Urban River Under Particular Interest For Recreational Use And As A Raw Water Source For Drinking Water: The Collaborative Research Project ``Safe Ruhr,Ä≥ - Microbiological Aspects | Strathmann et al. | *International Journal Of Hygiene And Environmental Health* | 2016 |
| The Road To Indigenous Extinction: Case Study Of Resource Exportation, Disease Importation, And Human Rights Violations Against The Urarina In The Peruvian Amazon. | Witzig and Acencios | *Health Hum Rights* | 1999 |
| The Role Of Birds In Dissemination Of Human Waterborne Enteropathogens | Graczyk et al. | *Trends In Parasitology* | 2008 |
| The Role Of Protected Areas In Supporting Human Health: A Call To Broaden The Assessment Of Conservation Outcomes | Terraube et al. | *Current Opinion In Environmental Sustainability* | 2017 |
| The Role Of Wetland Microinvertebrates In Spreading Human Diseases | Neogi et al. | *Wetlands Ecology And Management* | 2014 |
| The Spread Of Invasive Species And Infectious Disease As Drivers Of Ecosystem Change | Crowl et al. | *Frontiers In Ecology And The Environment* | 2008 |
| The Unintended Ecological And Social Impacts Of Food Safety Regulations In California's Central Coast Region | Karp et al. | *Bioscience* | 2015 |
| Threatened Plant Resources: Distribution And Ecosystem Services In The World'S High Elevation Park Of The Karakoram Ranges | Shedayi et al. | *Pakistan Journal Of Botany* | 2016 |

| | | | |
|---|---|---|---|
| Toward Holistic Evaluation And Assessment: Linking Ecosystems And Human Well-Being For The Three Gorges Dam | Kittinger et al. | *Ecohealth* | 2009 |
| Toxoplasma Gondii, Source To Sea: Higher Contribution Of Domestic Felids To Terrestrial Parasite Loading Despite Lower Infection Prevalence | Vanwormer et al. | *Ecohealth* | 2013 |
| Transmission Ecology Of Echinococcus Multilocularis: What Are The Ranges Of Parasite Stability Among Various Host Communities In China? | Pleydell et al. | *Parasitology International* | 2006 |
| Transport And Fate Of Escherichia Coli In Sediment Layer And Water Column Of Basins On Active Construction Sites | Kunkel et al. | *Transactions Of The Asabe* | 2013 |
| Tropical Fish Importation From Third Countries: The Potential Risk Of Introducing Human And Aquatic Animal Pathogens | Manfrin et al. | *Risk Analysis In Aquatic Animal Health, Proceedings* | 2001 |
| Trypanosoma Cruzi Transmission Cycle Among Wild And Domestic Mammals In Three Areas Of Orally Transmitted Chagas Disease Outbreaks | Roque et al. | *American Journal Of Tropical Medicine And Hygiene* | 2008 |
| Tuberculosis Infection In Wildlife From The Ruaha Ecosystem Tanzania: Implications For Wildlife, Domestic Animals, And Human Health | Clifford et al. | *Epidemiology And Infection* | 2013 |
| Understanding Framings And Perceptions Of Spillover Preventing Future Outbreaks Of Bat-Borne Zoonoses | Lawson et al. | *Disaster Prevention And Management* | 2017 |
| Understanding Wastewater Treatment Mechanisms: A Review On Detection, Removal, And Purification Efficiencies Of Faecal Bacteria Indicators Across Constructed Wetlands | Donde and Xiao | *Environmental Reviews* | 2017 |
| Unhealthy Landscapes: Policy Recommendations On Land Use Change And Infectious Disease Emergence. | Patz et al. | *Environ Health Perspect* | 2004 |
| Urban Health And Ecology: The Promise Of An Avian Biomonitoring Tool | Pollack et al. | *Current Zoology* | 2017 |
| Urbanized White Ibises (Eudocimus Albus) As Carriers Of Salmonella Enterica Of Significance To Public Health And Wildlife | Hernandez et al. | *Plos One* | 2016 |
| Using Chemical, Microbial And Fluorescence Techniques To Understand Contaminant Sources And Pathways To Wetlands In A Conservation Site | Rhymes et al. | *Science Of The Total Environment* | 2015 |
| Using Human Disease Outbreaks As A Guide To Multilevel Ecosystem Interventions. | Cook et al. | *Environ Health Perspect* | 2004 |
| Using Innovation To Address Hiv, Aids, And Environment Links: Intervention Case Studies From Zimbabwe, Uganda, And Malawi | De Souza et al. | *Population And Environment* | 2008 |
| Vaccination In Conservation Medicine | Plumb et al. | *Revue Scientifique Et Technique-Office* | 2007 |

| | | | |
|---|---|---|---|
| | | *International Des Epizooties* | |
| Valuing Conservation Benefits Of Disease Control In Wildlife: A Choice Experiment Approach To Bovine Tuberculosis Management In New Zealand'S Native Forests | Tait et al. | *Journal Of Environmental Management* | 2017 |
| Vertebrates Used For Medicinal Purposes By Members Of The Nyishi And Galo Tribes In Arunachal Pradesh (North-East India) | Chakravorty et al. | *Journal Of Ethnobiology And Ethnomedicine* | 2011 |
| Vertical Stratification Of Adult Mosquitoes (Diptera: Culicidae) Within A Tropical Rainforest In Sabah, Malaysia | Brant et al. | *Malaria Journal* | 2016 |
| Virus In Nonhuman Primates: Zoonosis, Anthroponosis And Biodiversity | Pujol | *Interciencia* | 2006 |
| Wastewater Irrigation And Environmental Health: Implications For Water Governance And Public Policy | Hanjra et al. | *International Journal Of Hygiene And Environmental Health* | 2012 |
| Watersheds, Forests, And Childhood Health: Global Relationships And Policy Opportunities | Ricketts et al. | *The Lancet* | 2017 |
| West Nile Virus And Wildlife | Marra et al. | *Bioscience* | 2004 |
| West Nile Virus Revisited: Consequences For North American Ecology | LaDeau et al. | *Bioscience* | 2008 |
| Wetlands And Human Health: An Overview | Dale and Connelly | *Wetlands Ecology And Management* | 2012 |
| Wetlands And Invertebrate Disease Hosts: Are We Asking For Trouble? | Malan et al. | *Water Sa* | 2009 |
| When Bats Go Viral: Negative Framings In Virological Research Imperil Bat Conservation | López-Baucells et al. | *Mammal Review* | 2018 |
| Wild Animal Conservation And Welfare In Agricultural Systems | Mathews | *Animal Welfare* | 2010 |
| Wild Primate Populations In Emerging Infectious Disease Research: The Missing Link? | Wolfe et al. | *Emerging Infectious Diseases* | 1998 |
| Wildlife And Pastoral Society - Shifting Paradigms In Disease Control | Kock et al. | *Domestic Animal/Wildlife Interface: Issue For Disease Control, Conservation, Sustainable Food Production, And Emerging Diseases* | 2002 |
| Wildlife Management Agencies Should Participate In Rabies Control | MacInnes and LeBer | *Wildlife Society Bulletin* | 2000 |
| Wildlife Trade And Human Health In Lao Pdr: An Assessment Of The Zoonotic Disease Risk In Markets | Greatorex et al. | *Plos One* | 2016 |
| Wildlife Tuberculosis In South African Conservation Areas: Implications And Challenges | Michel et al. | *Veterinary Microbiology* | 2006 |
| Woodland Biodiversity Management As A Tool For Reducing Human Exposure To Ixodes Ricinus Ticks: A Preliminary Study In An English Woodland | Medlock et al. | *Journal Of Vector Ecology* | 2015 |

| | | | |
|---|---|---|---|
| Zoonoses In Wildlife: Integrating Ecology Into Management | Mathews | *Advances In Parasitology* | 2009 |
| Zoonotic Disease Risk And The Bushmeat Trade: Assessing Awareness Among Hunters And Traders In Sierra Leone | Subramanian | *Ecohealth* | 2012 |
| Zoonotic Protozoa: From Land To Sea | Fayer et al. | *Trends In Parasitology* | 2004 |
| Zoonotic Schistosomiasis In Non-Human Primates: Past, Present And Future Activities At The Human-Wildlife Interface In Africa | Standley et al. | *Journal Of Helminthology* | 2012 |
